# Genome Mining and Metabolic Profiling Uncover Polycyclic Tetramate Macrolactams from *Streptomyces koyangensis* SCSIO 5802

**DOI:** 10.3390/md19080440

**Published:** 2021-07-31

**Authors:** Wenjuan Ding, Jiajia Tu, Huaran Zhang, Xiaoyi Wei, Jianhua Ju, Qinglian Li

**Affiliations:** 1 CAS Key Laboratory of Tropical Marine Bio-Resources and Ecology, Guangdong Key Laboratory of Marine Materia Medica, RNAM Center for Marine Microbiology, South China Sea Institute of Oceanology, Chinese Academy of Sciences, Guangzhou 510301, China; dingwenjbrb@foxmail.com (W.D.); tujyang@163.com (J.T.); aifyui@126.com (H.Z.); 2 College of Oceanology, University of Chinese Academy of Sciences, Beijing 100049, China; 3 Key Laboratory of Plant Resources Conservation and Sustainable Utilization, South China Botanical Garden, Chinese Academy of Sciences, Guangzhou 510650, China; weixiaoy@scib.ac.cn; 4 Key Special Project for Introduced Talents Team of Southern Marine Science and Engineering Guangdong Laboratory, Guangzhou 510301, China

**Keywords:** genome sequencing, gene disruption, polycyclic tetramate macrolactams, metabolic engineering, genome mining

## Abstract

We have previously shown deep-sea-derived *Streptomyces koyangensis* SCSIO 5802 to produce two types of active secondary metabolites, abyssomicins and candicidins. Here, we report the complete genome sequence of *S. koyangensis* SCSIO 5802 employing bioinformatics to highlight its potential to produce at least 21 categories of natural products. In order to mine novel natural products, the production of two polycyclic tetramate macrolactams (PTMs), the known 10-*epi*-HSAF (**1**) and a new compound, koyanamide A (**2**), was stimulated via inactivation of the abyssomicin and candicidin biosynthetic machineries. Detailed bioinformatics analyses revealed a PKS/NRPS gene cluster, containing 6 open reading frames (ORFs) and spanning ~16 kb of contiguous genomic DNA, as the putative PTM biosynthetic gene cluster (BGC) (termed herein *sko*). We furthermore demonstrate, via gene disruption experiments, that the *sko* cluster encodes the biosynthesis of 10-*epi*-HSAF and koyanamide A. Finally, we propose a plausible biosynthetic pathway to 10-*epi*-HSAF and koyanamide A. In total, this study demonstrates an effective approach to cryptic BGC activation enabling the discovery of new bioactive metabolites; genome mining and metabolic profiling methods play key roles in this strategy.

## 1. Introduction

Microbially produced natural products (NPs) play an important role in drug discovery, especially with respect to initiatives aimed at developing new agents for combatting infectious disease [1]. Increasing numbers and expanding structural diversity of new bioactive NPs identified from marine-derived *Streptomyces* suggest the increasing importance of marine-derived microbes as an important reservoir for new NPs [2]. Additionally, the rapid development of whole-genome sequencing technology has made it clear that microorganisms have great, though previously underappreciated potential to produce new bioactive NPs by virtue of biosynthetic gene clusters (BGCs) that remain “silent” under standard laboratory culture conditions [3]. For instance, around a dozen types of secondary metabolites had been discovered from the model organism *Streptomyces coelicolor* A3(2) before the publication of its genome sequence in 2002; however, seven additional metabolites were discovered by genome mining [4]. In light of this realization, genome mining has become a powerful tool by which to identify BGCs of interest which might then be activated to produce NPs of interest [5]. Indeed, many strategies to identify new natural products have benefitted from genome mining [6]; these include OSMAC (one strain-many compounds) [7], heterologous BGC expression [8], as well as manipulation of global or biosynthetic pathway-specific regulator strategies [9]. Recently, we investigated genetic inactivation of the biosynthetic genes of a microbe’s predominant products as a means generating new otherwise absent NPs. This approach also employs genome mining and has been demonstrated to be a good strategy to direct metabolic flux towards other biosynthetic routes, thus activating a silent gene cluster. For instance, genetic inactivation of the biosynthetic genes responsible for rishirilide and xiamycin production in *Streptomyces olivaceus* SCSIO T05 activated the production of lobophorin CR4 [10]. In another example, the production of nocardamine [11] and atratumycin [12] in *Streptomyces atratus* SCSIO ZH16 was achieved by inactivation of ilamycin biosynthesis. 

Polycyclic tetramate macrolactams (PTMs) are a unique class of natural products with a variety of valuable biological activities such as antibiotic, antifungal, antiprotozoal, and antitumor properties [13]. PTMs consist of a tetramate-containing macrocyclic lactam ring and a polycyclic carbocycle with a 5/5, 5/6, 5/5/6, or 5/6/5 ring system [14] (Figure S1). Multiple asymmetric centers in the PTMs greatly enhance their structural diversity and contribute to a wide range of biological activities [13,15,16]. The significant biological activities and intriguing chemical structures of PTMs have attracted a great deal of interest from synthetic and biosynthetic chemists. However, owing to the multiple stereocenters in representatives of the family, biosynthesis has thus far proven the most cost effective and efficient means of accessing the PTM scaffold. Detailed analysis of the genome sequence of the deep-sea-derived *Streptomyces koyangensis* SCSIO 5802 revealed the presence of at least 21 gene clusters including one putative PTM-encoding gene cluster. Previous chemical investigations of this strain have enabled the discovery of two types of active secondary metabolites, abyssomicins and candicidins; however, the PTM representatives have not been detected. In this paper, to activate the PTM gene cluster, the biosynthetic gene clusters for abyssomicin/candicidin were genetically inactivated. As a result, two PTMs, the known 10-*epi*-HSAF (**1**) and a new compound, koyanamide A (**2**), were isolated from the large scale fermentation of the mutant strain and their structures were unambiguously determined by extensive analyses of HRESIMS, 1D and 2D NMR, electronic circular dichroism (ECD) spectroscopic data. In addition, we also performed detailed analysis of the PTM gene cluster and proposed a biosynthetic pathway of PTMs in *S. koyangensis* SCSIO 5802.

## 2. Results and Discussion

### 2.1. Genome Features and Annotation of the Strain S. koyangensis SCSIO 5802

**Figure 1:**
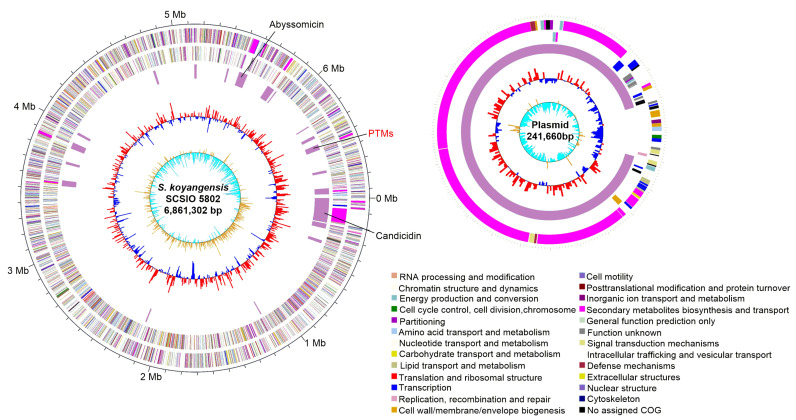
The complete genome of *S. koyangensis* SCSIO 5802. The five circles (from outer to inner) represent CDSs on the forward strand, CDSs on the reverse strand, nomenclature and locations of predictive secondary metabolite clusters, GC content, and GC skew. Plasmid contains one secondary metabolite BGC. The CDSs are colored according to the main COGs functional classification categories.

Whole genome sequencing is an important tool in efforts to discover new NPs [10,17]. To understand the full biosynthetic landscape of *S. koyangensis* SCSIO 5802, the microbe’s complete genome has been sequenced in previous study [18]. We reported and analyzed the genome data in this study. The SCSIO 5802 genome consists of a 6,861,302-bp circular chromosome and a 241,660-bp circular plasmid with an average G + C content of 73.29%. This complete genome was predicted to contain 6,419 protein-coding genes, 21 rRNA genes, and 65 tRNA genes (Figure 1 and Table 1).

To identify putative gene clusters within the SCSIO 5802 genome, the complete sequence was interrogated for secondary metabolite BGCs using online antiSMASH software [19]. The analysis revealed that a total of 21 putative gene clusters are contained within the SCSIO 5802 genome, including three polyketide synthase (PKS, Type I and Type III), four nonribosomal peptide synthetase (NRPS), two hybrid PKS-NRPS, three terpene, two bacteriocin, three saccharide, one lanthipeptide BGCs and two other categories of BGCs (Table 2). This finding suggests that SCSIO 5802 has the potential to produce at least 21 categories of NPs. Among the 21 predicted BGCs, cluster 18 was proposed to be involved in the biosynthesis of PTMs, since it shows high similarity to previously reported PTM gene clusters. However, only two types of secondary metabolites, abyssomicins and candicidins (Figure S2), have been isolated to date [20,21,22]. Thus, these analyses suggested that *S. koyangensis* SCSIO 5802 represented an outstanding candidate strain to mine PTM-type compounds.

### 2.2. Visualization of New NPs Produced by a Genetically Engineered Mutant 

**Figure 2:**
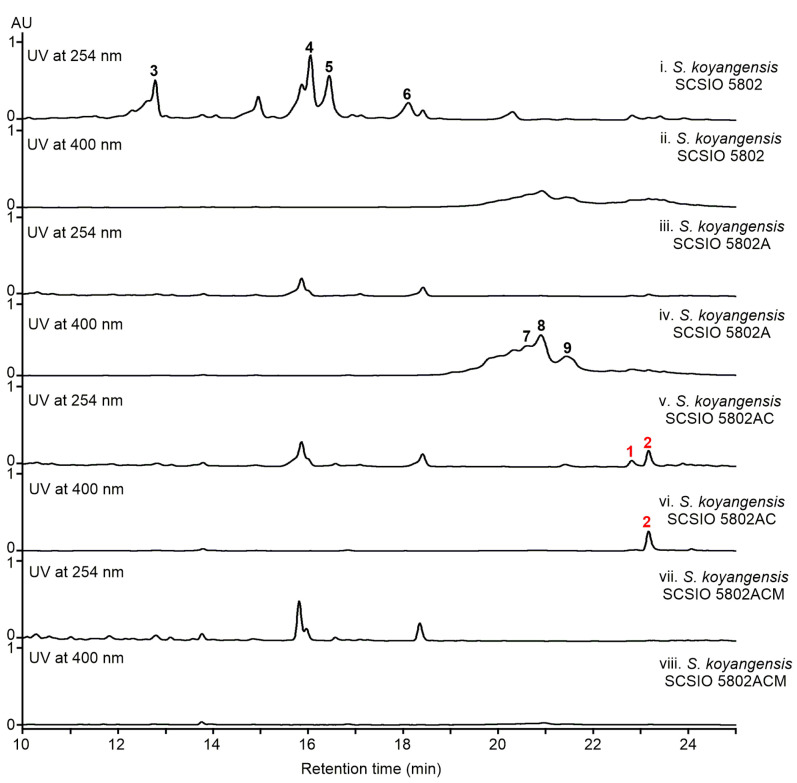
HPLC-based analyses of fermentation broths: (i–ii) *S. koyangensis* SCSIO 5802; (iii–iv) *S. koyangensis* SCSIO 5802A; (v–vi) *S. koyangensis* SCSIO 5802AC; (vii–viii) *S. koyangensis* SCSIO 5802ACM. Due to differences in the UV absorption wavelengths between **1**–**6** and **7**–**9**, all chromatograms employed UV detections at both 254 nm and 400 nm. Compounds **1** and **2** are 10-*epi*-HSAF and koyanamide A. Compounds **3**–**9** were previously identified as abyssomicin 4, neoabyssomicin B, abyssomicin 2, neoabyssomicin A, candicidin A3, candicidin D, and candicidin A1, respectively.

*S. koyangensis* SCSIO 5802 predominantly produces abyssomicins (**3**–**6**) under standard laboratory culture conditions [20,21] (Figure 2, traces i–ii). We hypothesized that this large metabolic background might obscure the presence of other, less abundant secondary metabolites. In our previous study, we had characterized AbmB1 as the PKS responsible for the assembly of the abyssomicin polyketide backbone. To enhance both the presence and detectability of other secondary metabolites, we have therefore generated an in-frame deletion mutant designated *S. koyangensis* SCSIO 5802A, devoid of the β-ketoacyl synthase (KS) and acyl transferase (AT) domains of *abmB1* [18]. HPLC analyses have revealed that the SCSIO 5802A mutant fails to produce abyssomicin [18]. Instead, the SCSIO 5802A mutant generated the polyenic antibiotics, candicidins (**7–9**) [22,23,24], as the major metabolites (Figure 2, traces iii–iv). To further minimize the metabolic background of the genetically engineered mutant *S. koyangensis* SCSIO 5802A, 1.8 kb of the PKS gene *canD* [22] responsible for the construction of the polyketide chain of candicidins was deleted using the in-frame format, thus affording the in-frame “double-deletion” mutant designated *S. koyangensis* SCSIO 5802AC (Figure S3). HPLC analyses of the fermentation extract revealed that this genetically engineered strain produced neither the candicidins nor the abyssomicins (Figure 2, traces v–vi). Notably, a group of secondary metabolites with UV spectra distinct from those of the abyssomicins and candicidins now constituted the major metabolites produced by *S. koyangensis* SCSIO 5802AC. The ablation of both candicidin and abyssomicin biosynthesis enabled the production and visualization of these newly produced compounds in quantities sufficient for structure elucidation.

*S. koyangensis* SCSIO 5802 predominantly produces abyssomicins (**3**–**6**) under standard laboratory culture conditions [20,21] (Figure 2, traces i–ii). We hypothesized that this large metabolic background might obscure the presence of other, less abundant secondary metabolites. In our previous study, we had characterized AbmB1 as the PKS responsible for the assembly of the abyssomicin polyketide backbone. To enhance both the presence and detectability of other secondary metabolites, we have therefore generated an in-frame deletion mutant designated *S. koyangensis* SCSIO 5802A, devoid of the β-ketoacyl synthase (KS) and acyl transferase (AT) domains of *abmB1* [18]. HPLC analyses have revealed that the SCSIO 5802A mutant fails to produce abyssomicin [18]. Instead, the SCSIO 5802A mutant generated the polyenic antibiotics, candicidins (**7–9**) [22,23,24], as the major metabolites (Figure 2, traces iii–iv). To further minimize the metabolic background of the genetically engineered mutant *S. koyangensis* SCSIO 5802A, 1.8 kb of the PKS gene *canD* [22] responsible for the construction of the polyketide chain of candicidins was deleted using the in-frame format, thus affording the in-frame “double-deletion” mutant designated *S. koyangensis* SCSIO 5802AC (Figure S3). HPLC analyses of the fermentation extract revealed that this genetically engineered strain produced neither the candicidins nor the abyssomicins (Figure 2, traces v–vi). Notably, a group of secondary metabolites with UV spectra distinct from those of the abyssomicins and candicidins now constituted the major metabolites produced by *S. koyangensis* SCSIO 5802AC. The ablation of both candicidin and abyssomicin biosynthesis enabled the production and visualization of these newly produced compounds in quantities sufficient for structure elucidation.

### 2.3. Fermentation, Isolation, and Structural Elucidation of PTMs from S. koyangensis SCSIO 5802AC 

To elucidate the two newly generated NPs, a 21 L scale fermentation of the *S. koyangensis* SCSIO 5802AC mutant was performed with a two-step fermentation process as previously reported [20]. The culture broth was extracted with butanone, and the mycelial cake was extracted with acetone. Silica gel chromatography and RP-HPLC purification methods afforded analytically pure samples of **1** and **2**.

**Figure 3:**
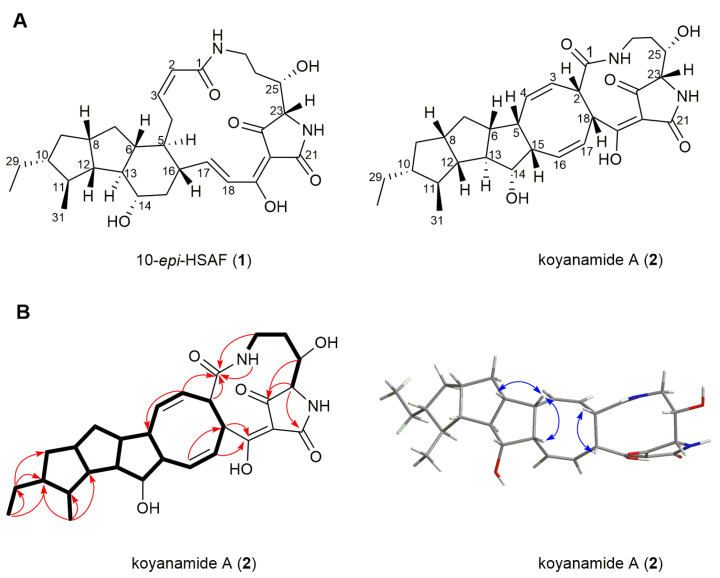
(**A**) Structures of the isolated 10-*epi*-HSAF (**1**) and koyanamide A (**2**); (**B**) selected COSY (bold), HMBC (arrows) and key NOESY correlations (blue arrows) for koyanamide A (**2**).

The molecular formula of **1** was determined as C_29_H_40_N_2_O_6_ by HRESIMS ([M + H]^+^, *m/z* 513.2967, calcd for 513.2973, Figure S4). The planar structure of **1** was determined to be the same as that of 10-*epi*-HSAF [25,26], on the basis of NMR spectroscopic data comparisons for **1** (Table S3; Figures S5–S11) and 10-*epi*-HSAF [25,26]. The relative configurations of **1** were assigned by NOESY correlations and then compared with 10-*epi*-HSAF [25,26]. Very obvious NOESY correlations of H-5/H-13, H-6/H-8, H-6/H-14, H-6/H-16, H-8/H-10, and H-10/H-12 were observed; H-10 and H-11 were assigned to be *trans*-oriented for **1**. In parallel, we also measured the electronic circular dichroism (ECD) spectrum of **1**, which was found to be in excellent agreement with the calculated ECD spectrum of 5*S*, 6*R*, 8*R*, 10*R*, 11*S*, 12*S*, 13*R*, 14*S*, 16*S*, 23*S*, 25*S* and 10-*epi*-HSAF (Figure S12). Thus, the structure of **1** was consistent with 10-*epi*-HSAF (Figure 3A) [25,26].

**Figure 4:**
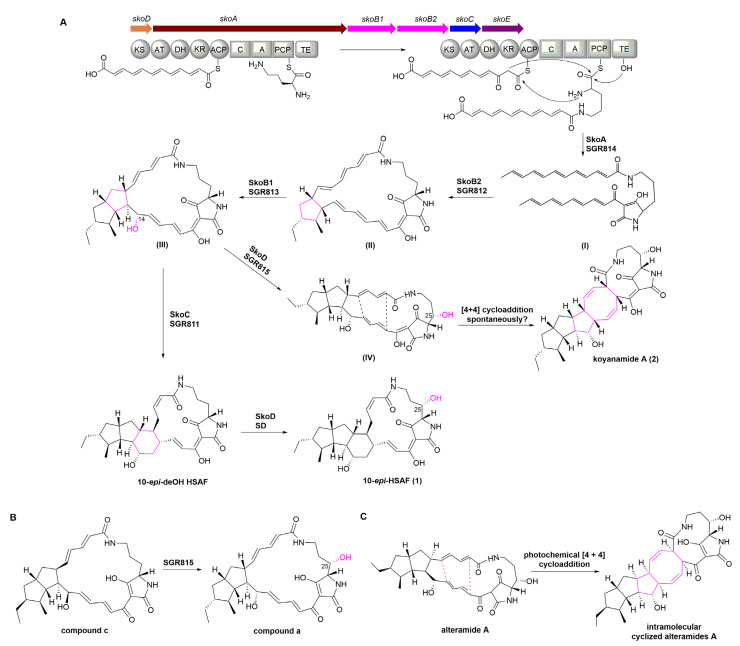
(**A**) Proposed biosynthetic pathway to 10-*epi*-HSAF (**1**) and koyanamide A (**2**). (**B**) SGR815 encoded in the *SGR810-815* cluster has been shown to hydroxylate the C25 position in compound c [28]. (**C**) The photochemical [4 + 4] cycloaddition converting alteramide A to intramolecularly cyclized alteramide A has been confirmed [29].

The molecular formula of **2** was determined to be C_29_H_38_N_2_O_6_ on the basis of HRESIMS ([M + H]^+^, *m/z* 511.2810, calcd for 511.2815, Figure S13). A comprehensive analysis of the NMR data for **2** revealed that the structure of **2** was similar to that of **1** (Figures S14–S20). A careful comparison suggested that the C2–C3 double bond (*δ*_H_ 5.70 and 5.89; *δ*_C_ 124.1 and 139.1) and C17–C18 double bond (*δ*_H_ 6.57 and 6.86; *δ*_C_ 150.2 and 121.3) in **1** were absent in **2**; instead, the C3–C4 double bond (*δ*_H_ 5.46 and 5.56; *δ*_C_ 125.9 and 138.3) and C16–C17 double bond (*δ*_H_ 5.75 and 5.67; *δ*_C_ 138.2 and 126.1) were found in **2**. The coupling constants (*J*_3,4_ = 9.1 Hz and *J*_16,17_ = 8.8 Hz) indicated that these double bonds are in the *Z* configurations. The ^1^H–^1^H COSY spectrum of **2** indicated the presence of carbon–carbon bonds from C2 to C18 and from C5 to C15 (Figure 3B). Thus, we deduced that **2** contains a cyclooctadiene unit. The rest of the NMR data for both **1** and **2** were almost identical. These analyses suggested that **2** is likely derived from intermediate **IV** via an intramolecular photochemical [4 + 4] cycloaddition, as has been reported for alteramide A (Figure 4A,C) [27]. From a biosynthetic perspective, absolute configurations of the first two five-membered rings, C14, C23 and C25 in **2** should be the same as those found in **1**. The absolute configurations of the four remaining stereogenic centers at C2, C5, C15 and C18 in **2** were established with the use of NOESY spectra; correlations of H-2/H-18, H-5/H-15, and H-5/H-6 allowed the determination of the absolute configurations at these centers to be as 2*S*, 5*S*, 15*S*, 18*S* (Figure 3B). Thus, compound **2** was named koyanamide A.

### 2.4. Bioinformatic Analysis of the PTM Gene Cluster and the Proposed Biosynthetic Pathway of PTMs in S. koyangensis SCSIO 5802

**Figure 5:**
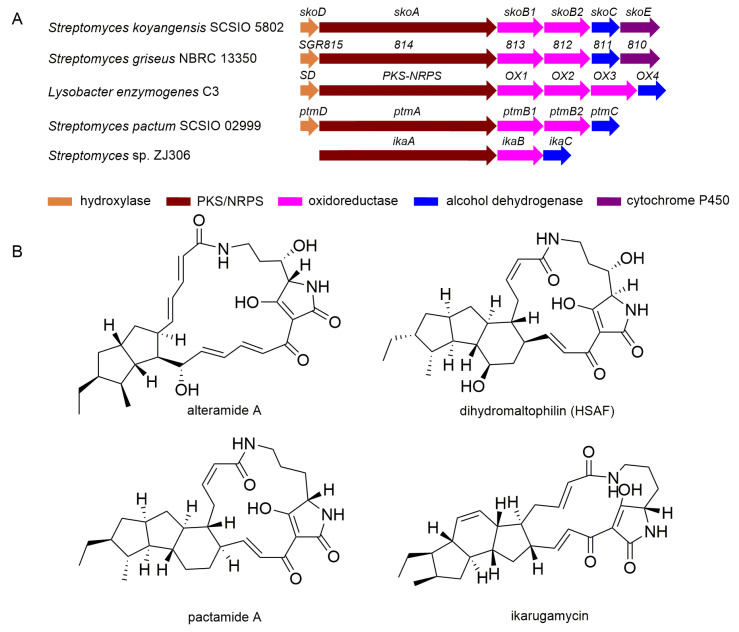
(**A**) Comparative analysis of sko gene cluster with other reported PTM BGCs, SGR810-815 from Streptomyces griseus NBRC 13350 [28], HSAF BGC from Lysobacter enzymogenes C3 [30], ptm gene cluster from Streptomyces pactum SCSIO 02999 [8], ika gene cluster from Streptomyces sp. ZJ306 [31]; (**B**) the corresponding structures of PTMs whose biosyntheses are encoded by clusters indicated in panel A.

Bioinformatic analysis revealed that cluster 18 (termed the *sko* gene cluster) was proposed to be the gene cluster responsible for the biosynthesis of compounds **1** and **2**, since it shows high similarity to previously reported PTM gene clusters, such as *SGR810-815* BGC from *Streptomyces griseus* [28] (Figure 5). The *sko* cluster was found to contain six genes including a hybrid PKS/NRPS gene *skoA*, two FAD-dependent oxidoreductases genes *skoB1* and *skoB2*, an alcohol dehydrogenase *skoC*, a sterol desaturase (SD) gene *skoD*, and a cytochrome P450 gene *skoE* (Figure 5 and Table 3).

The center of the gene cluster is the hybrid PKS/NRPS encoding gene *skoA*; SkoA comprises one PKS module and one NRPS module, making up a total of nine domains (KS-AT-DH-KR-ACP-C-A-PCP-TE). As reported, this type of hybrid PKS/NRPS is con-served among PTM producing bacterial species [32] (Figure 5). The single-module PKS of the hybrid PKS/NRPS enzyme is iteratively used to produce two separate polyketide chains. These chains are, respectively, linked with the α- and δ- amino groups of an l-ornithine that is tethered in the NRPS portion to generate a common polyene tetramate intermediate [25,26]. SkoA shows high similarity to the previously characterized PKS/NRPS SGR814 (identity/similarity, 71%/79%) encoded in the SGR810-815 BGC from *Streptomyces griseus* and the PKS/NRPS enzyme (identity/similarity, 62%/73%) encoded within the HSAF cluster from *Lysobacter enzymogenes* C3 (Table 3). Accordingly, SkoA is proposed to be responsible for the assembly of the polyene tetramate precursor in the biosynthetic pathway to compounds **1** and **2.**

Downstream of the *skoA* are two oxidoreductases encoding genes (*skoB1* and *skoB2*). SkoB1 and SkoB2 strongly correlate to SGR813 (similarity/identity, 82%/89%) and SGR812 (similarity/identity, 84%/90%) enzymes encoded by the *SGR810-815* gene cluster, respectively. It has been demonstrated that formation of the diverse carbocyclic ring in PTMs, after formation of the tetramate polyene precursor, is catalyzed by a set of oxidoreductases. Generally, the presence of a single FAD-dependent oxidoreductase (such as IkaB in the ikarugamycin biosynthetic pathway) is suggested to catalyze the formation of the 5/6 ring system in PTMs [31]. In contrast, the presence of two or three oxidoreductases (such as SGR812 and SGR813 in the alteramide A biosynthetic pathway, OX1–OX3 [30] in HSAF biosynthesis, as well as PtmB1 and PtmB2 [8] in pactamide A assembly) is thought necessary for the formation of 5/5 ring systems [25] (Figure 5). SGR812 or PtmB2 or OX3 was demonstrated to catalyze the formation of the first five-membered ring [8,30], whereas the formation of the second five-membered ring appeared to be catalyzed by SGR813 or PtmB1 or OX1/OX2 [8,30]. Notably, it has been confirmed that formation of the second ring is coupled to C14 hydroxylation during HSAF biosynthesis [33]. The presence of two oxidoreductases in the *sko* gene cluster is consistent with the 5/5 ring system seen in **1** and **2**. Thus, SkoB2 is predicted to catalyze the formation of the first five-membered ring, whereas SkoB1 is proposed to install the second five-membered ring coupled with C14 hydroxylation *en route* to **1** and **2**.

Right flanking of *skoB1* and *skoB2* is the gene for alcohol dehydrogenase (SkoC); this enzyme shows high similarity to IkaC (similarity/identity, 61%/73%) in the ikarugamycin system, OX4 (similarity/identity, 68%/80%) encoded in the HSAF gene cluster, SGR811 (similarity/identity, 78%/84%) encoded in the *SGR810-815* gene cluster, and PtmC (similarity/identity, 70%/79%) encoded in the *ptm* gene cluster. IkaC and SGR11/OX4 have been demonstrated to install the inner five- or six-membered rings, respectively, of the scaffold via a Michael addition reaction [31,34]. Therefore, SkoC is proposed to catalyze the formation of the inner six-membered ring seen in compounds **1** and **2**.

Flanking the left of the PKS/NRPS encoding *skoA* is a sterol desaturase (SD) gene, *skoD*. SkoD corresponds well to the SD found encoded in the HSAF gene cluster [35], SGR815 encoded in *SGR810-815* gene cluster (Figure 5, Table 3) and FtdA encoded in the frontalamide gene cluster [32]. The SD and FtdA have been demonstrated to be responsible for the C25-hydroxylation in the corresponding 5/5/6 ring system [32,35]; SGR815 has been confirmed to hydroxylate C25 of the 5/5 ring system. Importantly, such hydroxylases have been shown to exhibit a reasonable amount of substrate promiscuity [32,36]. Thus, SkoD is proposed to catalyze the C25-hydroxylation of two different substrates in the biosynthetic pathway to compounds **1** and **2.**

To the right of the *sko* gene cluster is encoded a cytochrome P450 enzyme (SkoE); this enzyme shows high similarity to PtmE’(similarity/identity, 71%/77%) encoded in the *ptm’* gene cluster and FtdF (similarity/identity, 60%/72%) encoded in the frontalamide cluster. PtmE’ was predicted to catalyze hydroxylation at C12 whereas FtdF was predicted to catalyze 14-dehydrogenation [26,32]; however, neither of these proposed functions have been experimentally verified thus far.

Based on the above bioinformatics analysis of gene functions and organization of the *sko* gene cluster, we propose putative biosynthetic pathways leading to 10-*epi*-HSAF (**1**) and compound **2** (Figure 4A). We envision the following route to assembly for both **1** and **2**. First, iterative catalysis of the single-module PKS of SkoA produces two separate polyketide chains. The NRPS portion of SkoA then catalyzes the formation of two amide bonds employing the α and δ-amino groups of the same amino acid (ornithine) via two Claisen condensations and the subsequent formation of the tetramate moiety via a Dieckmann-type reaction to generate a polyene tetramate precursor **I** (Figure 4A). The FAD-dependent oxidoreductase SkoB2 catalyzes formation of the cyclopentane ring in **II**, and **II** is then acted upon by SkoB1 to install the second five-membered ring of the fused 5/5 system while also installing the C14 OH moiety to afford intermediate **III**. Subsequently, the alcohol dehydrogenase SkoC catalyzes formation of the six-membered necessary to yield 10-*epi*-deOH-HSAF. Finally, the sterol desaturase SkoD catalyzes hydroxylation at C25 of 10-*epi*-deOH-HSAF to form 10-*epi*-HSAF (**1**). Meanwhile, due to the functional promiscuity of SkoD as repoted for other characterized sterol desaturases (e.g., SGR815 [28]) (Figure 4B), SkoD may also hydroxylate C25 of intermediate **III** to generate intermediate **IV**. Intermediate **IV** might be further transformed into the hexacyclic **2** spontaneously as reported for altermide A [29] (Figure 4C) and combamide C [37]. It has been reported that alteramide A is susceptible to a unique intramolecular photochemical [4 + 4] cycloaddition to generate hexacyclic products [29]. Similar spontaneous reactions have also been proposed in the biosynthesis of combamide C [37]. Notably, cytochrome P450 SkoE showed high similarity with FtdF; however, the function of FtdF has not been experimentally verified thus far. Based on the inspiration of P450 BvnD [38], which was reported to set the stage for the following spontaneous [4 + 2] Diels–Alder reaction, we speculated that SkoE might be involved in accelerating the spontaneous [4 + 4] cycloaddition; however, this hypothesis requires further study.

To demonstrate the validity of the putative *sko* gene cluster in the biosynthesis of 10-*epi-*HSAF (**1**) and koyanamide A (**2**), the 1.6 kb KS domain coding region in the PKS/NRPS gene *skoA* was deleted using PCR-targeting methods; the resulting mutant strain, *S. koyangensis* SCSIO 5802ACM, was verified by both phenotypic and genotypic means (Figure S21). Fermentation and extraction of the SCSIO 5802ACM strain was carried out enabling follow up metabolomics. Not surprisingly, the HPLC-based metabolite analyses of the SCSIO 5802ACM mutant fermentation revealed the complete absence of **1** and **2**, thereby confirming the indispensability of the *sko* BGC for the biosynthesis of compounds **1** and **2** (Figure 2, traces vii–viii).

## 3. Materials and Methods

### 3.1. General Experimental Procedures

All bacterial strains and plasmids used in this study are listed in supplementary file 1: Tables S1 and S2, respectively. We have described the culture conditions for *S. koyangensis* SCSIO 5802 in previous study [20]. We have also previously described the culture conditions for *Escherichia coli,* including DH5α, BW25113/pIJ790, ET12567/pUZ8002 [39]. The medium was added with antibiotics, when necessary, at the following concentrations: chloramphenicol (Chl) 25 μg/mL, kanamycin (Kan) 50 μg/mL, apramycin (Apr) 50 μg/mL, and trimethoprim (TMP) 50 μg/mL.

A Chirascan circular dichroism spectrometer (Applied Photophysics, Ltd., Surrey, UK) was used to measure the CD spectra. The 1D and 2D NMR spectra were obtained using a Bruker AV-700 MHz NMR spectrometer (Bruker Biospin GmbH, Rheinstetten, Germany). Mass spectral data were determined using a quadrupole-time-of-flight mass spectrometry (Bruker Maxis 4G, Billerica, MA, USA). Column chromatography (CC) was performed using silica gel (100–200 mesh, Jiangyou Silica gel development, Inc., Yantai, China) and Sephadex LH-20 (GE Healthcare Bio-Sciences AB, Uppsala, Sweden). HPLC was carried out using a reversed-phase column (Phenomenex Gemini C18, 250 mm × 4.6 mm, 5 µm; Phenomenex, Torrance, CA, USA). Semi-preparative HPLC was performed with Hitachi HPLC station (Hitachi-L2130, Hitachi, Tokyo, Japan), a Diode Array Detector (Hitachi L-2455, Hitachi, Tokyo, Japan) and a Phenomenex ODS column (250 mm × 10 mm, 5 μm; Phenomenex, Torrance, CA, USA).

### 3.2. Genome Sequencing, Annotation, and Analysis

Sequencing of the complete genome was accomplished in the prior study [18]. The complete chromosome and plasmid sequences for SCSIO 5802 have been deposited in the GenBank database with accession numbers CP049945 and CP049946, respectively. Genes involved in secondary metabolic pathways were predicted using online antiSMASH software (http://antismash.secondarymetabolites.org/). The deduced ORFs were analyzed using online FramePlot 4.0beta software (http://nocardia.nih.go.jp/fp4/, accessed on 5 July 2021) and their functional predictions were accomplished with an online BLAST program (http://blast.ncbi.nlm.nih.gov/, accessed on 5 July 2021). The PKS architectures were analyzed using an NRPS-PKS online website (http://nrps.igs.umaryland.edu/nrps/, accessed on 5 July 2021). The identity and similarity values between proteins encoded in *sko* gene cluster and their homolog proteins were analyzed using an online Protein BLAST program (https://blast.ncbi.nlm.nih.gov/Blast.cgi?PROGRAM=blastp, accessed on 5 July 2021).

### 3.3. Construction of an In-Frame “Double-Deletion” Mutant SCSIO 5802AC and Inactivation of skoA in SCSIO 5802AC 

In-frame gene deletions were achieved by following the REDIRECT protocol [40]. The *S. koyangensis* SCSIO 5802 genomic cosmid library was constructed as previously [18]. The apramycin resistance gene *oriT-aac(3)IV* fragment was obtained by using specific primers (Table S1) that contain additional *Spe*I restriction sites, and used to replace the target gene *can* in the cosmid 7–8F. Restriction digest of the mutant cosmid with *Spe*I and subsequent relegation predictably abolished the apramycin resistance gene *oriT*-*aac(3)IV* fragment. The second round of PCR-targeting was performed to replace the kanamycin resistance gene on SuperCos I with another apramycin resistance gene *oriT-aac(3)IV* fragment obtained by a primer pair ARK [41]. The constructed mutant cosmid was introduced into non-methylating *Escherichia coli* ET12567/pUZ8002 and then transferred into *S. koyangensis* SCSIO 5802A, an in-frame deletion mutant, which has been constructed previously [22]. The single-crossover mutant strain was selected from antibiotic selection (apramycin^R^). To obtain the double-crossover mutant strain, the single-crossover mutant strain was first cultured on antibiotic-free ISP-4 medium. The double-crossover mutant was then selected by showing the apramycin^S^ phenotypes. The double-crossover mutant was finally confirmed by PCR verification (Figure S3). The in-frame “double-deletion” mutant was designated SCSIO 5802AC. 

The inactivation of *skoA* in SCSIO 5802AC was performed by λ-RED-mediated PCR-targeting mutagenesis method. Cosmid 6–8A was introduced into *E. coli* BW25113/pIJ790 to inactivate *skoA*. The *oriT-aac(3)IV* cassette was amplified by PCR from pIJ773 using primers skoA-delF and skoA-delR (Table S1), and introduced into *E. coli* BW25113/pIJ790/cosmid 6–8A to replace *skoA* via λ-RED-mediated recombination. Correct recombination was established by PCR using primers skoA-testF and skoA-testR. The mutated cosmid was then introduced into *E. coli* ET12567/pUZ8002 for further conjugation with SCSIO 5802AC. The double crossover mutant was obtained by antibiotic selection (Apr^R^Kan^S^) and confirmed by PCR using primers skoA-testF and skoA-testR (Figure S21), which was designated SCSIO 5802ACM.

### 3.4. Secondary Metabolites Analyses of S. koyangensis SCSIO 5802 and Related Derivative Strains 

The wild-type *S. koyangensis* SCSIO 5802 and relevant gene-inactivated mutants were first grown on A1 medium agar [20] at 28° C for 4–5 days to achieve sporulation. A portion of mycelium and spores (1 cm^2^) for each strain was added to 250 mL flasks containing 50 mL of RA medium. Fermentations were then carried out at 28 °C on rotary shakers (200 rpm) for 8 days. After fermentation, each fermentation culture was washed with 100 mL butanone, and the butanone solvent was removed under reduced pressure to afford an oily residue. Residues were each dissolved into 1 mL MeOH and centrifuged at 13,000× *g* for 10 min; supernatants were subjected to HPLC–UV analyses, each of which was performed using an Agilent Technologies 1260 Infinity system using a Phenomenex ODS column (150 × 4.6 mm, 5 μm), eluting with a linear gradient of 5 to 65% solvent B (solvent B: CH_3_CN + 0.1% trifluoroacetic acid (TFA); solvent A: H_2_O + 0.1% TFA) over 20min, followed by 65% to 100% solvent B in 2 min, and then 100% solvent B for 5 min, at a flow rate of 1 mL/min. Since the UV absorption wavelengths of compounds **1**–**6** and compounds **7**–**9** are different, chromatograms employed UV detection at both 254 nm and 400 nm to ensure comprehensive detection of new compounds.

### 3.5. Production, Isolation, and Structure Elucidation of 10-epi-HSAF (***1***) and koyanamide A (***2***)

To obtain compound **1** and **2**, large-scale fermentation (21 L) of the *S. koyangensis* SCSIO 5802AC was carried out. Both fermentation procedures were the same as that employed for *S. koyangensis* SCSIO 5802 wild type [20]. The extract was subjected to silica gel CC using gradient elution with a CHCl_3_/MeOH mixture (100:0, 98:2, 96:4, 94:6, 92:8, 90:10, 80:20, 70:30 and 50:50) to give 9 fractions (AFr.1-AFr.9). AFr.7 containing compound **1–2** were purified by preparative HPLC with an ODS column, eluted with 40% solvent B to 80% solvent B (solvent A: H_2_O + 0.1% TFA; solvent B: CH_3_CN + 0.1% TFA) over a period of 30 min at a flow rate of 2.5 mL/min (using detection at 254 nm), to afford compound **1** (20 mg), compound **2** (14 mg). The purified compounds **1** and **2** were subjected to MS, ^1^H, and ^13^C NMR spectra measurements and elucidated as 10-*epi*-HSAF and a new compound koyanamide A (**2**), respectively.

## 4. Conclusions

In this study, we analyzed the complete genome of *S. koyangensis* SCSIO 5802 and employed antiSMASH to unveil 21 putative BGCs including one putative PTM-encoding gene cluster. Ablation of the abyssomicin and candicidin biosynthetic pathways [22], major products of SCSIO 5802, enabled us to see two previously absent peaks in HPLC analyses of fermentation extracts from the *S. koyangensis* SCSIO 5802AC mutant strain. The two new HPLC signals were isolated and identified as PTM-type compounds, 10-*epi*-HSAF (**1**) and koyanamide A (**2**). Within the genome of SCSIO 5802 we also were able, using bioinformatics, to identify the *sko* BGC responsible for the production of **1** and **2**; gene inactivation experiments verified **1** and **2** as direct products of the *sko* biosynthetic machinery. Finally, enabled by our knowledge of the full *sko* cluster and structural elucidations of **1** and **2**, we have formulated a cogent biosynthetic pathway leading to these otherwise unobserved NPs. This work highlights metabolic engineering and genome mining as effective tools by which to turn on putatively silent BGCs; the result is expanded NP chemical diversity for drug discovery initiatives.

## Figures and Tables

**Table 1:** General Genomic Features of *S. koyangensis* SCSIO 5802.

Features	Chromosome	Plasmid
Genome topology	circular	circular
Assembly size (bp)	6,861,301	241,660
G + C content (%)	73.29	
Protein coding genes	6419	
tRNA genes	65	
rRNA genes	21	
Secondary metabolite gene clusters	20	1
GenBank accession	CP049945	CP049946

**Table 2:** Putative BGCs for *S. koyangensis* SCSIO 5802 as predicted by AntiSMASH.

BGC	Position		Type (Product)
	From	To	
Cluster 1	1425	204,688	NRPS, T1PKS, NRPS-like (Candicidin)
Cluster 2	212,498	254,791	T3PKS, betalactone (Herboxidiene)
Cluster 3	354,622	380,374	Terpene, bacteriocin (Isorenieratene)
Cluster 4	1,148,592	1,158,990	Ectoine (Ectoine)
Cluster 5	2,121,247	2,133,190	Siderophore (Desferrioxamine B)
Cluster 6	3,525,814	3,575,656	NRPS (Gobichelin)
Cluster 7	3,709,353	3,731,640	Lassopeptide (Kanamycin)
Cluster 8	3,844,037	3,898,083	NRPS (Mannopeptimycin)
Cluster 9	3,967,931	3,989,806	Lanthipeptide (SAL-2242)
Cluster 10	4,795,477	4,815,134	Terpene (Albaflavenone)
Cluster 11	5,138,618	5,159,926	Terpene (Kanamycin)
Cluster 12	5,411,522	5,426,592	Siderophore (Ficellomycin)
Cluster 13	5,513,113	5,586,859	T1PKS (Abyssomicin)
Cluster 14	5,772,383	5,834,468	NRPS, T1PKS (Kanamycin)
Cluster 15	5,850,776	5,860,672	Bacteriocin
Cluster 16	6,243,324	6,253,539	Bacteriocin
Cluster 17	6,332,792	6,358,781	Terpene (Hopene)
Cluster 18	6,410,409	6,458,458	NRPS, T1PKS (PTMs)
Cluster 19	6,633,149	6,655,731	Terpene
Cluster 20	6,781,885	6,832,651	NRPS, T1PKS, Lantipeptide (Oxazolonycin)
Cluster 21	10,602	230,749	Butyrolactone, T1PKS (Stambomycin)

**Table 3:** Deduced functions of open reading frames (ORFs) in the *sko* BGC.

ORF	Size ^a^	Proposed Function	HSAF Homolog; ID/SI (%)	SGR Homolog; ID/SI (%)
*skoD*	281	sterol desaturase	SD; 59/70	815; 76/82
*skoA*	3137	PKS/NRPS (KS-AT-DH-KR-ACP-C-A-PCP-TE)	PKS-NRPS; 62/73	814; 71/79
*sko* *B1*	560	FAD-dependent oxidoreductase	OX2; 69/81	813; 82/89
*sko* *B2*	576	FAD-dependent oxidoreductase	OX3; 75/87	812; 84/90
*sko* *C*	351	alcohol dehydrogenase	OX4; 68/80	811; 78/84
*sko* *E*	399	cytochrome P450	-	810; 71/77

^a^ Size in units of amino acids (aa); ID/SI: identity/similarity.

## Data Availability

The authors declare that all relevant data supporting the findings of this study are available within the article and its Supplementary Materials file, or from the corresponding authors upon request.

## Supplementary Materials

The following are available online at https://www.mdpi.com/article/10.3390/md19080440/s1, Table S1: Strains and plasmids used and constructed in this study; Table S2: Primers used in this study; Table S3: ^1^H (700 MHz) and ^13^C NMR (175 MHz) NMR data for 10-*epi*-HSAF (**1**) and koyanamide A (**2**); Figure S1: Structures of polycyclic tetramate macrolactam (PTM) antibiotics; Figure S2: Structures of abyssomicins/neoabyssomicins and candicidins isolated from *S. koyangensis* SCSIO 5802; Figure S3: *canD* disruption in *S. koyangensis* SCSIO 5802A via PCR-targeting; Figures S4–S11: HRESIMS and NMR spectra of compound **1**; Figure S12: Comparison among the ECD spectra measured for compound **1**, 10-*epi*-HSAF and calculated for compound **1**; Figures S13–S20: HRESIMS and NMR spectra of compound **2**; Figure S21: *sko**A* disruption in *S. koyangensis* SCSIO 5802AC via PCR-targeting.
