# Supplementary material for: Genome Mining and Metabolic Profiling Uncover Polycyclic Tetramate Macrolactams from Streptomyces koyangensis SCSIO 5802
Source: Mar Drugs. 2021 Jul 31;19(8):440. doi: 10.3390/md19080440 (PMC8399814; doi:10.3390/md19080440)
Supplement: Supplementary file 1 [file marinedrugs-19-00440-s001.zip › marinedrugs-1307368-supplementary.pdf]

Genome mining and metabolic profiling uncover polycyclic tetramate macrolactams from *Streptomyces koyangensis* SCSIO 5802

Wenjuan Ding [1,2], Jiajia Tu [1], Huaran Zhang [1,2], Xiaoyi Wei [4], Jianhua Ju [1,2,3,*] and Qinglian Li [1,3,*]

[1] CAS Key Laboratory of Tropical Marine Bio-Resources and Ecology, Guangdong Key Laboratory of Marine Materia Medica, RNAM Center for Marine Microbiology, South China Sea Institute of Oceanology, Chinese Academy of Sciences, Guangzhou 510301, China; 13760785354@163.com (W.D.); 1152492875@qq.com (J.T.)

[2] College of Oceanology, University of Chinese Academy of Sciences, Beijing 100049, China

[3] Key Special Project for Introduced Talents Team of Southern Marine Science and Engineering Guangdong Laboratory, Guangzhou 510301, China

[4] Key Laboratory of Plant Resources Conservation and Sustainable Utilization, South China Botanical Garden, Chinese Academy of Sciences, Guangzhou 510650, China

* Correspondence: liql@scsio.ac.cn (Q.L.); jju@scsio.ac.cn (J.J.); Tel.: +86-20-8902-3028

Supporting Information Table of Contents

Entry	Content	Page/s
**1.**	**Table S1.** Strains and plasmids used and constructed in this study.	3
**2.**	**Table S2.** Primers used in this study.	4
**3.**	**Table S3.** ^{1}H (700 MHz) and ^{13}C NMR (175 MHz) NMR Data for HSAF (**1**) and koyanamide A (**2**).	5
**4.**	**Figure S1.** Structures of polycyclic tetramate macrolactam (PTM) antibiotics.	6
**5.**	**Figure S2.** Structures of abyssomicins/neoabyssomicins and candicidins isolated from *S. koyangensis* SCSIO 5802.	7
**6.**	**Figure S3.** *canD* disruption in *S. koyangensis* SCSIO 5802A *via* PCR-targeting.	8
**7.**	**Figure S4.** HRESIMS spectrum of **1**.	9
**9.**	**Figure S5.** ^{1}H NMR spectrum of **1** in DMSO-*d6*.	10
**10.**	**Figure S6.** ^{13}C NMR spectrum of **1** in DMSO-*d6*.	11
**11.**	**Figure S7.** ^{13}C DEPT spectrum of **1** in DMSO-*d6*.	12
**12.**	**Figure S8.** ^{1}H-^{1}H COSY spectrum of **1** in MeOH-*d4*.	13
**13.**	**Figure S9.** HSQC spectrum of **1** in MeOH-*d4*.	14
**14.**	**Figure S10.** HSBC spectrum of **1** in MeOH-*d4*.	15
**15.**	**Figure S11.** NOESY spectrum of **1** in MeOH-*d4*.	16
**16**	**Figure S12.** Comparison among the ECD spectra measured for compound **1**, 10-*epi*-HSAF and calculated for **1**.	17
**17.**	**Figure S13.** HRESIMS spectrum of **2**.	18
**18.**	**Figure S14.** ^{1}H NMR spectrum of **2** in MeOH-*d4*.	19
**19.**	**Figure S15.** ^{13}C NMR spectrum of **2** in MeOH-*d4*.	20
**20.**	**Figure S16.** ^{13}C DEPT spectrum of **2** in MeOH-*d4*.	21
**21.**	**Figure S17.** ^{1}H-^{1}H COSY spectrum of **2** in MeOH-*d4*.	22
**22.**	**Figure S18.** HSQC spectrum of **2** in MeOH-*d4*.	23
**23.**	**Figure S19.** HSBC spectrum of **2** in MeOH-*d4*.	24
**24.**	**Figure S20.** NOESY spectrum of **2** in MeOH-*d4*.	25
**25.**	**Figure S21.** *skoA* disruption in *S. koyangensis* SCSIO 5802AC *via* PCR-targeting.	26
**26**	**Supplemental References.**	27

Table S1. Strains and plasmids used and constructed in this study.

**Strains or plasmids**	**Description**	**Reference or source**
**Strains**		
E. coli DH5α	Host for general cloning	
E. coli BW25113	Host strain for PCR-targeting	[1]
E. coli ET12567/pUZ8002	Host strain for conjugation	[2]
S. koyangensis SCSIO 5802	Wild-type producer of abyssomycins	This study
S. koyangensis SCSIO 5802A	Abyssomycins-free strain originated from *S. koyangensis* SCSIO 5802	This study
S. koyangensis SCSIO 5802AC	Abyssomycins/candicidins-free strain originated from *S. koyangensis* SCSIO 5802	This study
S. koyangensis SCSIO 5802ACM	Abyssomycins/candicidins/PTM-free strain originated from *S. koyangensis* SCSIO 5802	This study
**Plasmids**		
pIJ790	Cml^r, including λ-RED (*gam*, *bet*, *exo*) for PCR-targeting	[3]
pIJ773	Apr^r , source of *acc(3)IV* and *oriT* fragment	[3]
pUZ8002	Kan^r , including *tra* for conjugation	[4]
7-8F	Amp^r, Kan^r, harboring *can* gene	This study
6-8A	Amp^r, Kan^r, harboring *skoB* gene	This study

Table S2. Primers used in this study.

Primer	Sequence (5'-3')
**For PCR screening of genomic library**	
sko-orf5567-up-scr-F	GGGCAAGGTCGTCGTCA
sko-orf5567-up-scr-R	CCGTCGGAACCGTAGTTGA
sko-orf5573-mid-scr-F	CGGCACGATGATGACGATG
sko-orf5573-mid-scr-R	GCCAGTGCGGTCTTGATGAG
sko-orf5576-down-scr-F	AGCCGGGTCTACGAGAAGG
sko-orf5576-down-scr-R	TCCAGGAAGGTGGTGAGGG
**For construction of gene disruption mutant**	
canD-delF	GCGCGCGTCGAGGTGGGCGAGCAGCCGGGCGG CCTGGGCACTAGTATTCCGGGGATCCGTCGACC
canD-delR	GAGGTGCCCCCGCTCCTGCGCGGCCTGGTCCGC ACCCGCACTAGTTGTAGGCTGGAGCTGCTTC
skoA-delF	TTGGCTGACGCGATCAACGACGCCCCGACGGGC AAGGTAATTCCGGGGATCCGTCGACC
skoA-delR	CCAGACCAGACGCCGGTCCTCGGGCTCCAGCCG CCGTCCTGTAGGCTGGAGCTGCTTC
canD-testF	GCCAGCGAGTGGGTGACG
canD-testR	CGGCGAACTGTCCGAGCG
skoA-testF	CATCCCCTCCACCGCGCGAAG
skoA-testR	GACCGGTTCGCTCTCCAGG

Table S3. ^{1}H (700 MHz) and ^{13}C NMR (175 MHz) NMR Data for 10-*epi*-HSAF (**1**) and koyanamide A (**2**).

	10-*epi*-HSAF (**1**)[a]		koyanamide A (**2**)[b]	
position	δ_C, type	δ_H, mult (J in Hz)	δ_C, type	δ_H, mult (J in Hz)
1	165.5, C		180.7, C	
2	124.2, CH	5.74, m	46.6, CH	3.91, m
3	138.8, CH	5.91, m	125.9, CH	5.46, t (9.1)
4	27.8, CH_2	1.87-1.92, m; 3.44-3.45, m	138.3, CH	5.56, d (9.8)
5	43.6, CH	1.25-1.28, m	46.4, CH	2.92-2.95, m
6	47.2, CH	1.61-1.65, m	58.4, CH	1.89-1.92, m
7	37.3, CH_2	0.82-0.88, m; 1.98-2.06, m	36.5, CH_2	0.99-1.00, 1.96-1.98, m
8	41.4, CH	2.34, m	50.5, CH	2.79-2.81, m
9	40.5, CH_2	0.82-0.88, m; 1.98-2.06, m	41.6, CH_2	0.94-0.95, 2.10, m
10	53.5, CH	1.29-1.36,m	54.9, CH	1.38-1.39, m
11	46.5, CH	1.23-1.29, m	47.8, CH	1.25-1.28, m
12	58.0, CH	1.73-1.75, m	54.2, CH	1.67-1.72, m
13	59.2, CH	1.09-1.13, m	65.4, CH	1.83-1.86, m
14	72.7, CH	3.21-3.24, m	82.1, CH	3.67-3.68, m, 1H
15	42.2, CH_2	1.20-1.31, m; 1.73-1.75, m	59.6, CH	3.17-3.21, m
16	44.8, CH	1.98-2.06, m	138.2, CH	5.75, t (8.8)
17	148.8, CH	6.52, m	126.1, CH	5.67, t (8.7)
18	119.9, CH	6.79, m	47, CH	3.92, m
19	171.5, C		207.7, C	
20	100.5, C		–[c], C	
21	177.1, C		176.7 (Z),174.7 (E), C	
22		8.88, s		–[c]
23	68.4, CH	3.85, s	69.8, 69.9, CH	3.94-3.97,m
24	192.0, C		207.7, C	
25	69.9, CH	3.80, m	71.2 (Z),72.5 (E), CH	3.74-3.76, m
26	31.3, CH_2	1.17, m; 1.36-1.41, m	30.8, 31.3, CH_2	1.36, 1.29, m
27	36.3, CH_2	2.57, m; 3.21-3.24, m	37.2, CH_2	3.64-3.69, m, 2H
28		7.97, t (5.5)		–[c]
29	25.9, CH_2	1.01-1.02, m; 1.53-1.57, m	27.2, CH_2	1.1, 1.62-1.64, m
30	12.6, CH_3	0.85, m	13.0, CH_3	0.91, d (7.5)
31	18.4, CH_3	1.06, d (6.3)	18.4, CH_3	1.05, d (6.5)

[a]Measured in DMSO-d_6. [b]Measured in CD_3OD. [c]Not observed.

Figure S1. Structures of PTMs with a subset of 5/5 bicyclic ring system and 5/5/6 tricyclic ring system.

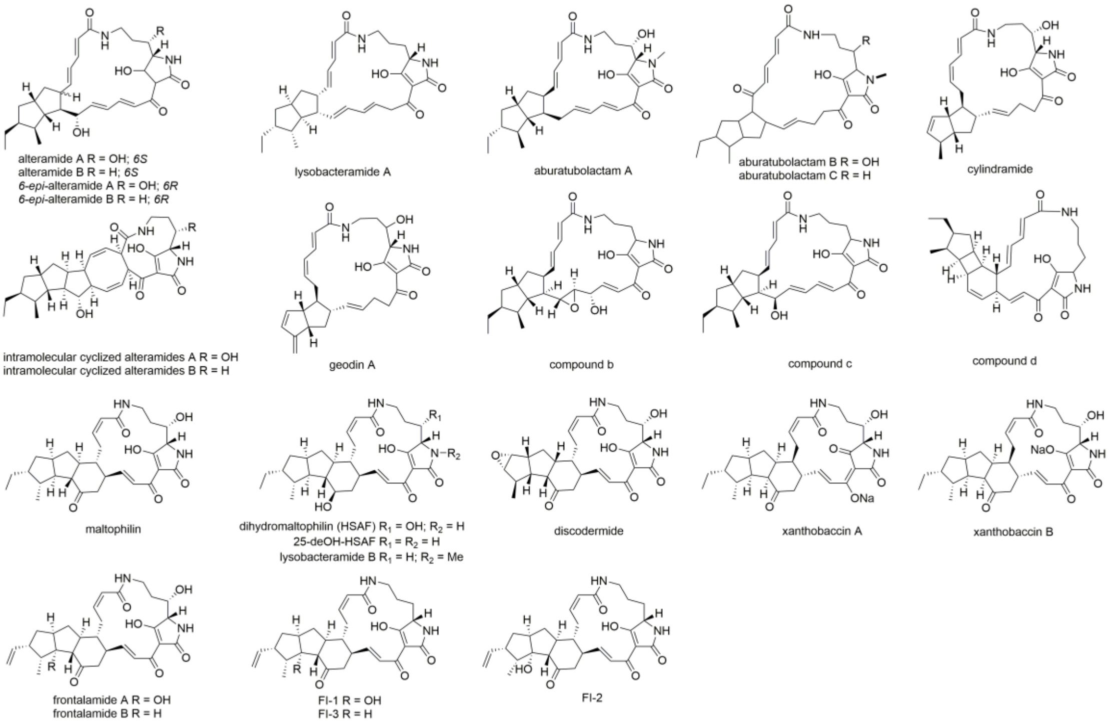

Figure S2. Structures of abyssomicins/neoabyssomicins and candicidins isolated from *S. koyangensis* SCSIO 5802.

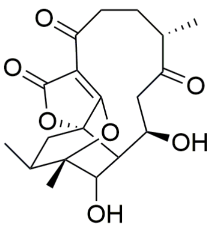

abyssomicin 4 (**3**)

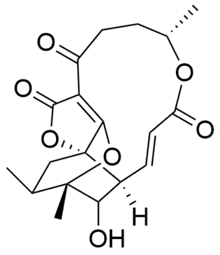

neoabyssomicin B (**4**)

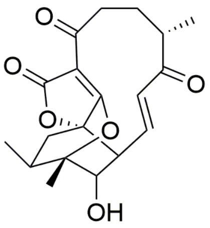

abyssomicin 2 (**5**)

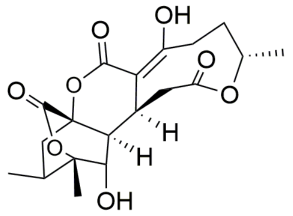

neoabyssomicin A (**6**)

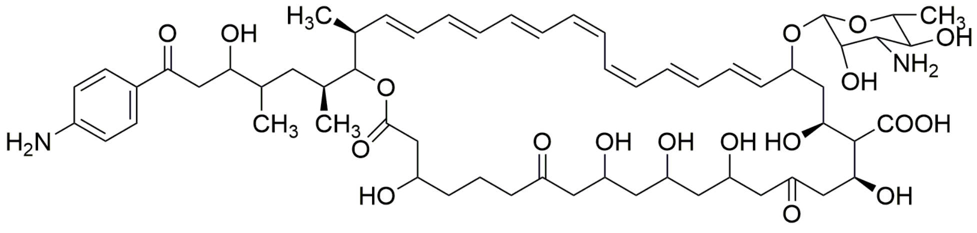

candicidin A3 (**7**)

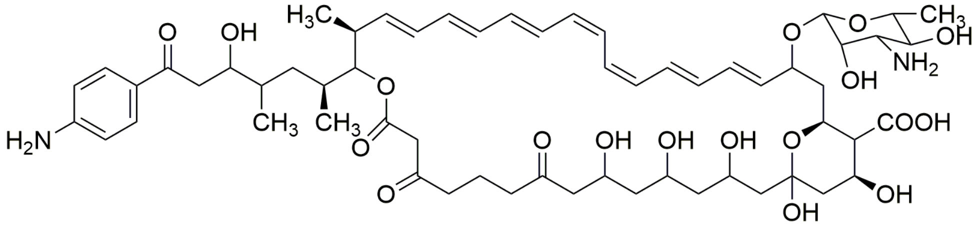

candicidin D (**8**)

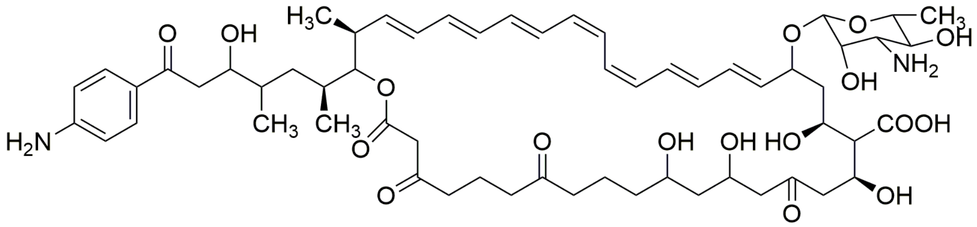

candicidin A1 (**9**)

Figure S3. *canD* disruption in *S. koyangensis* SCSIO 5802A *via* PCR-targeting.

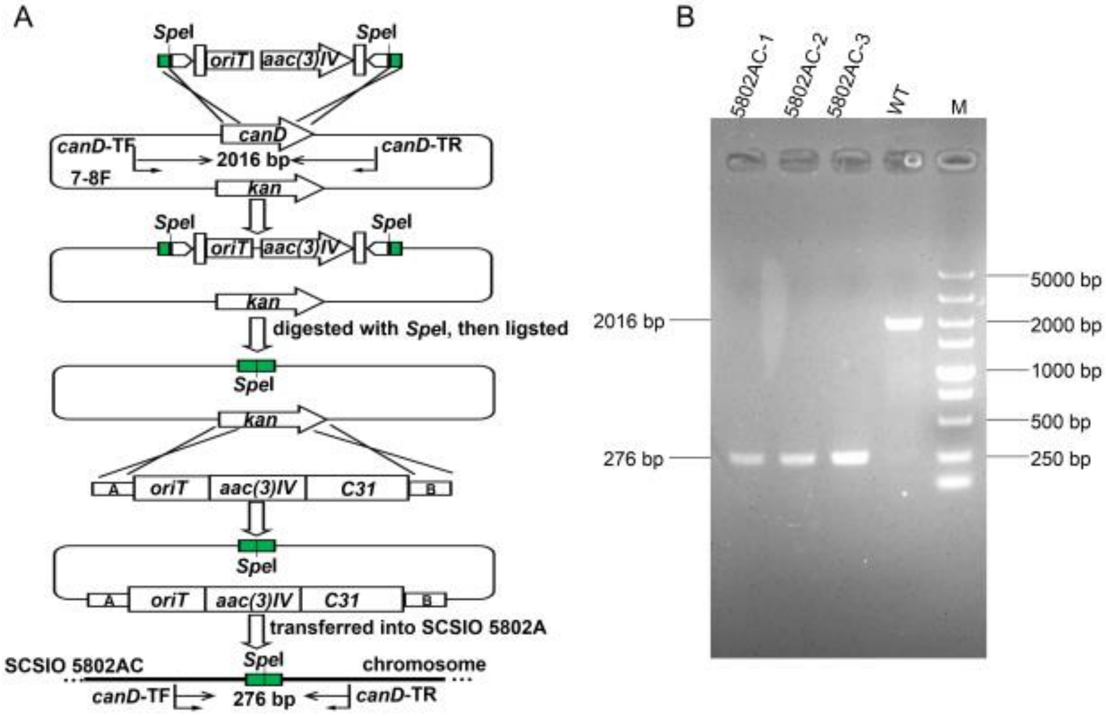

Figure S4. HRESIMS spectrum of compound **1**.

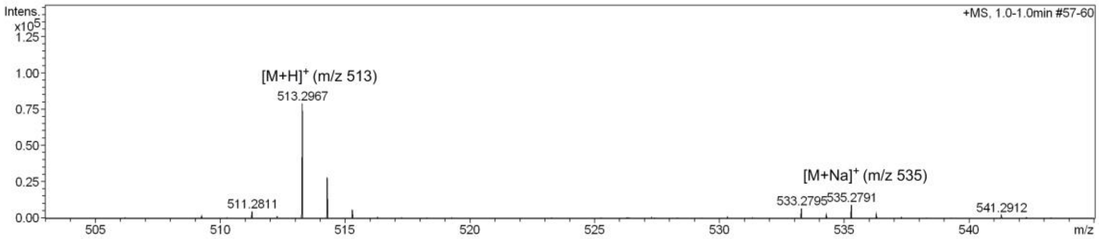

Meas. m/z	#	Ion Formula	Score	m/z	err [ppm]	err [mDa]	mSigma	rdb	e⁻ Conf	N-Rule
513.2967	1	C29H41N2O6	100.00	513.2959	-1.5	-0.8	17.1	10.5	even	ok
535.2791	1	C29H40N2NaO6	100.00	535.2779	2.3	1.2	9.9	10.5	even	ok
1025.5859	1	C58H81N4O12	100.00	1025.5846	-1.3	-1.4	22.8	20.5	even	ok
1047.5664	1	C58H80N4NaO12	100.00	1047.5665	0.1	0.1	70.0	20.5	even	ok

Figure S5. ^{1}H NMR spectrum of **1** in DMSO-*d6*.

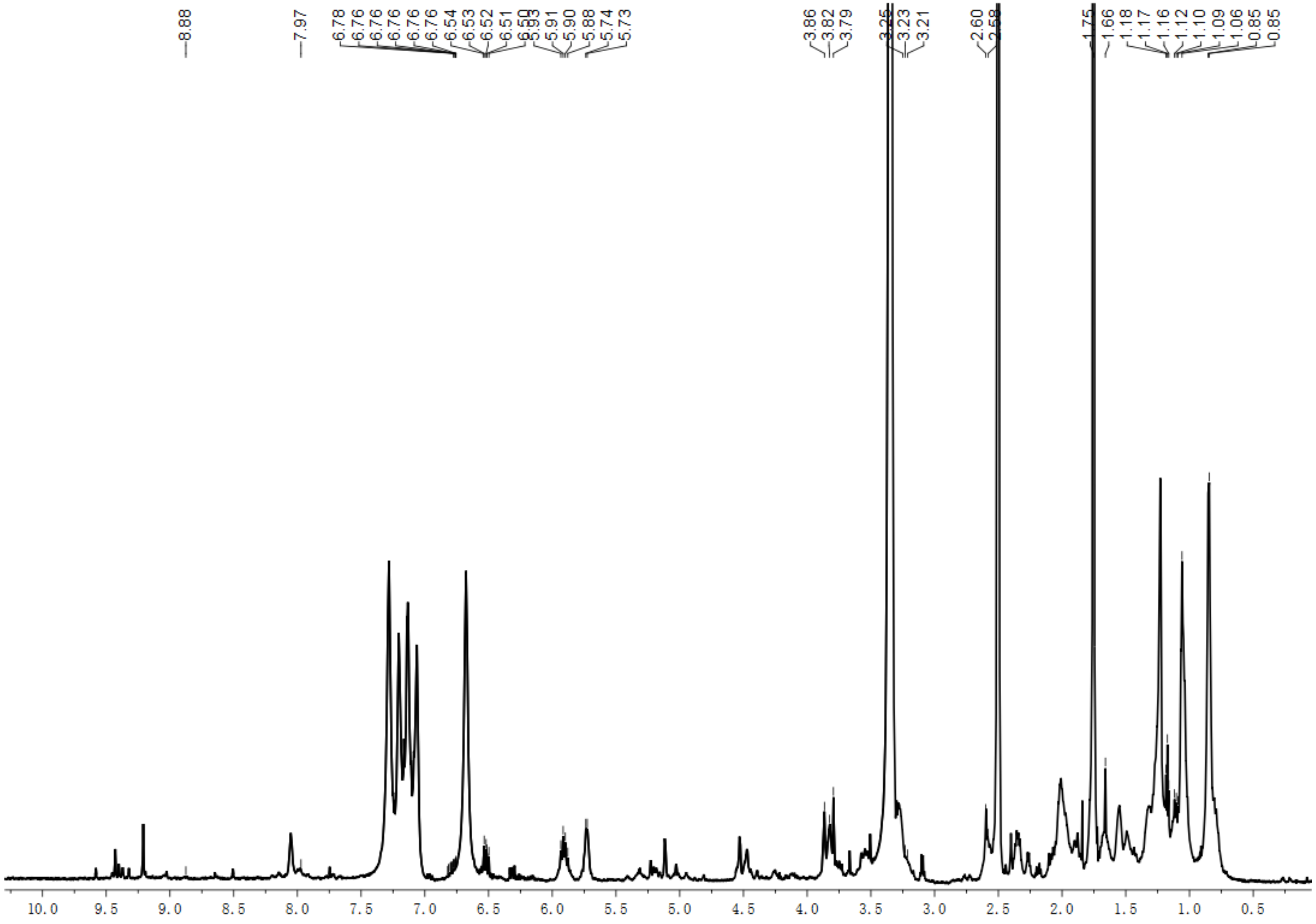

Figure S6. ^{13}C NMR spectrum of **1** in DMSO-*d6*.

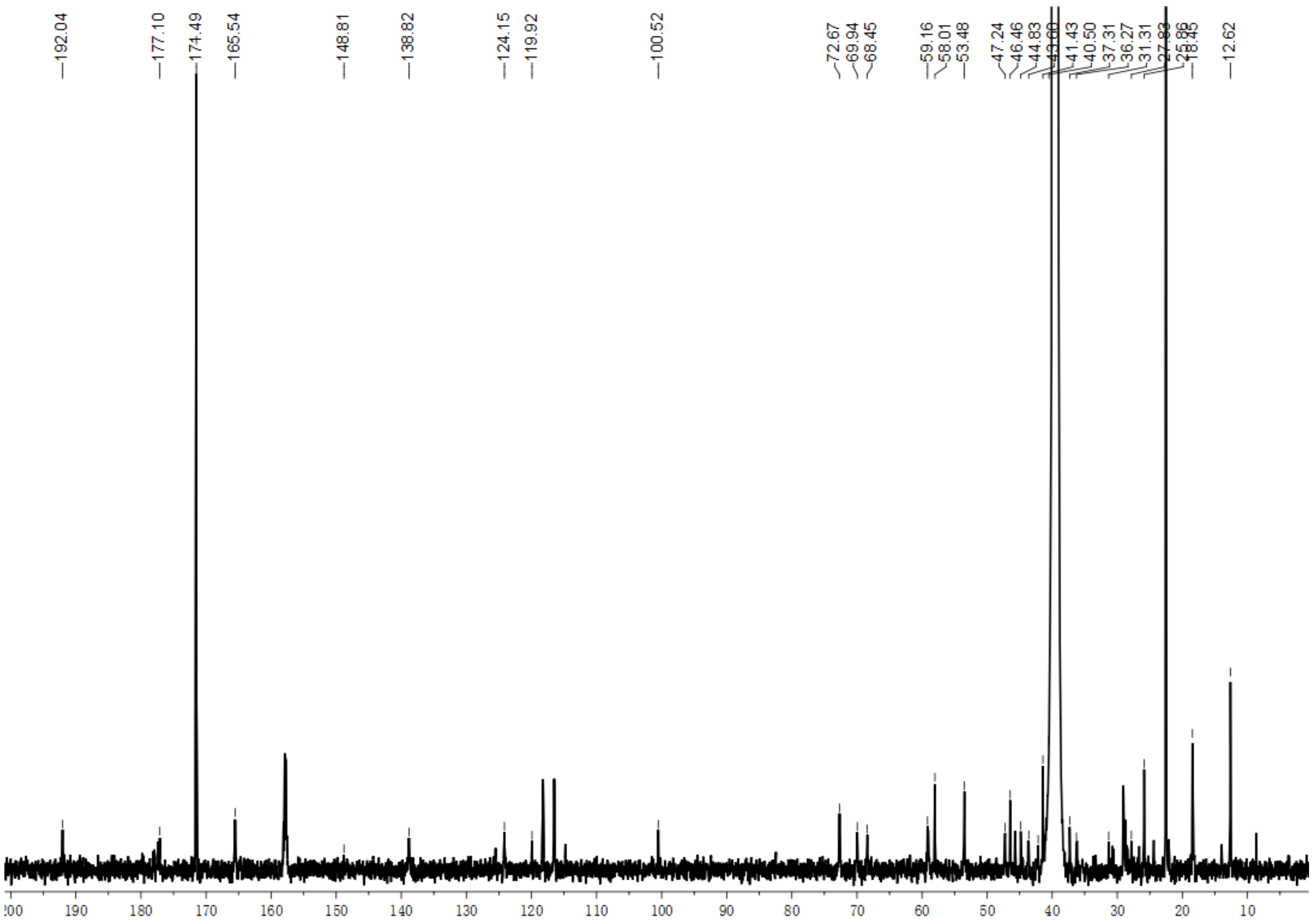

Figure S7. ^{13}C DEPT spectrum of **1** in DMSO-*d6*.

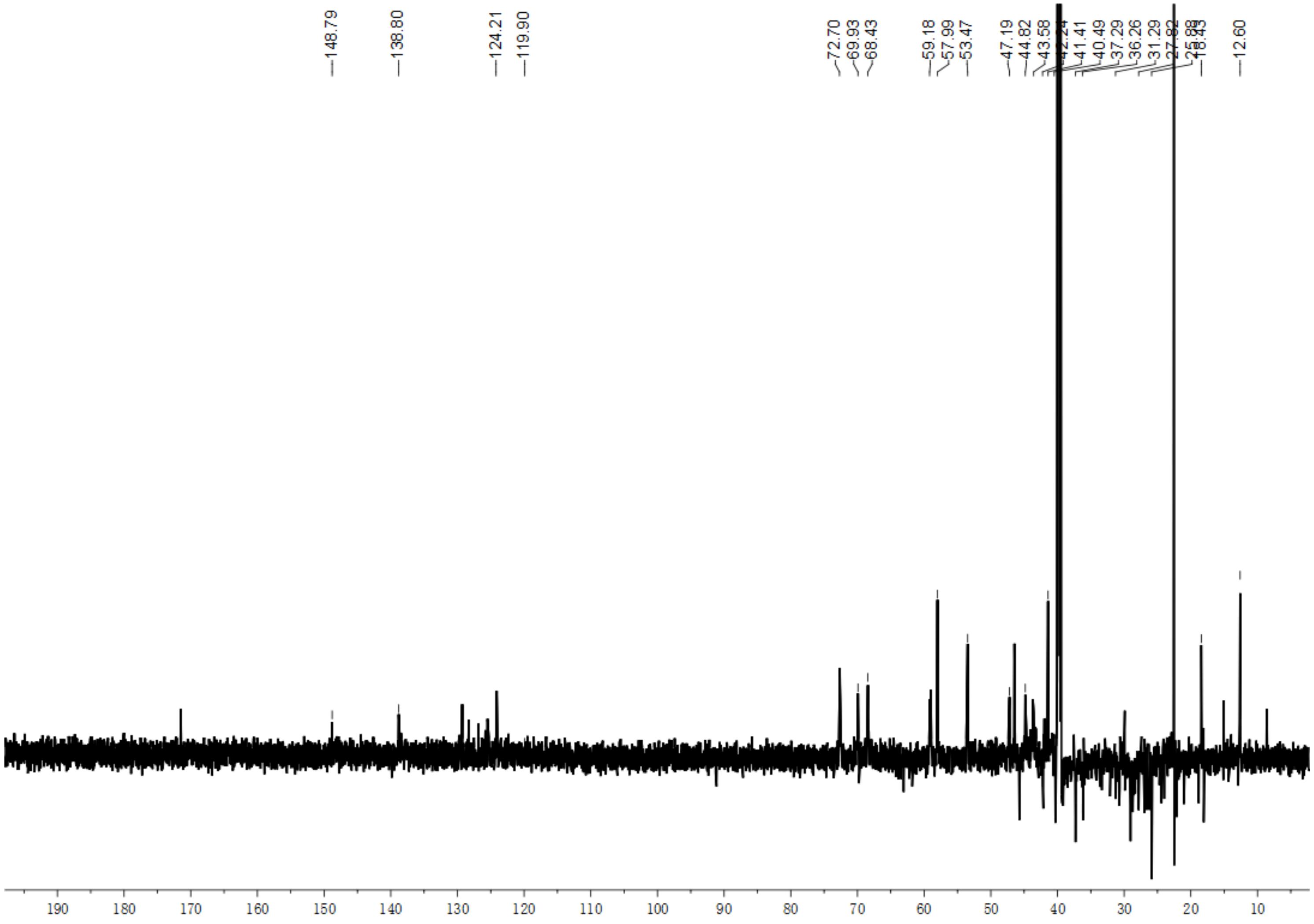

Figure S8. ^{1}H-^{1}H COSY spectrum of **1** in DMSO-*d6*.

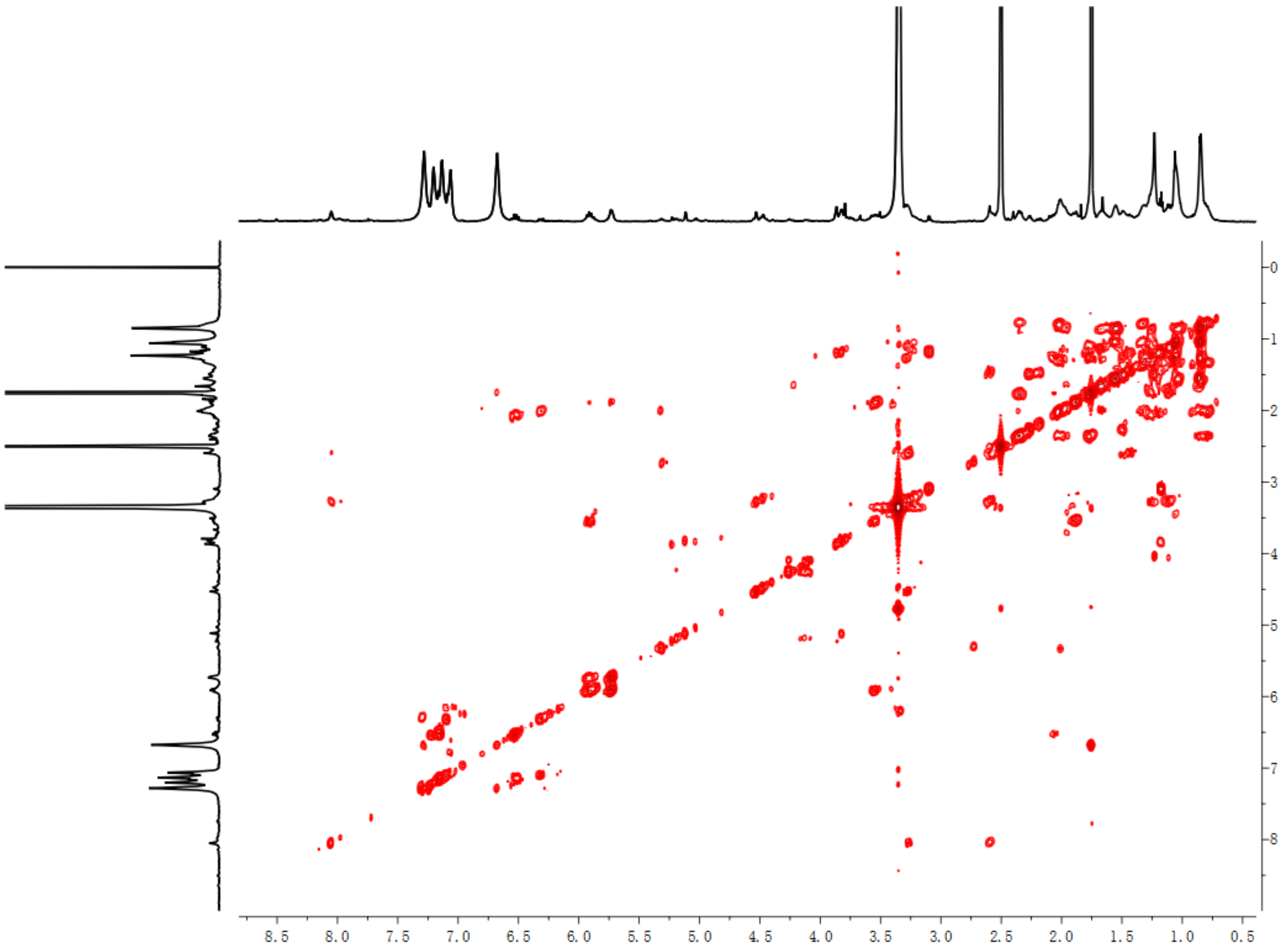

Figure S9. HSQC spectrum of **1** in DMSO-*d6*.

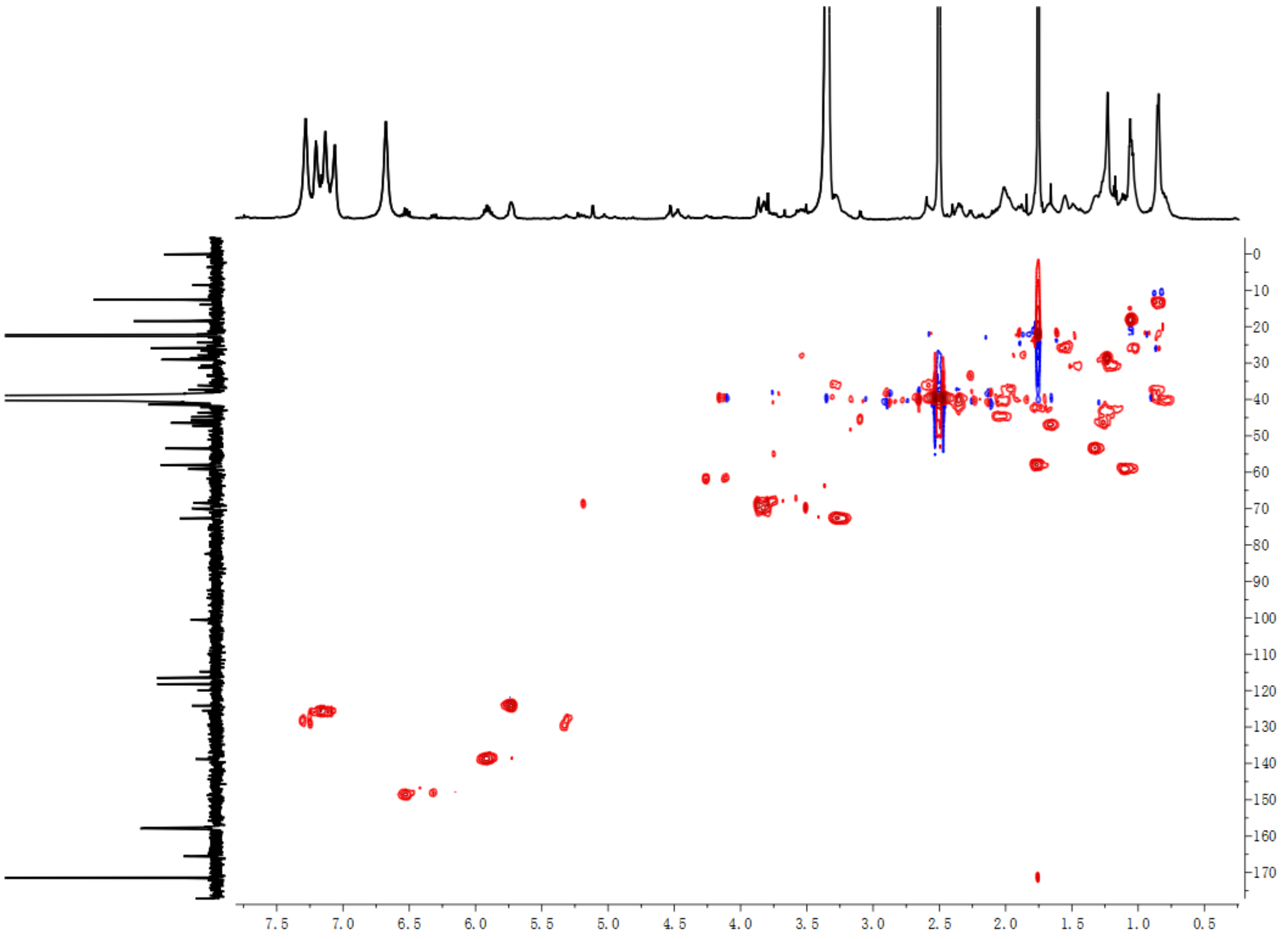

Figure S10. HSBC spectrum of **1** in DMSO-*d6*.

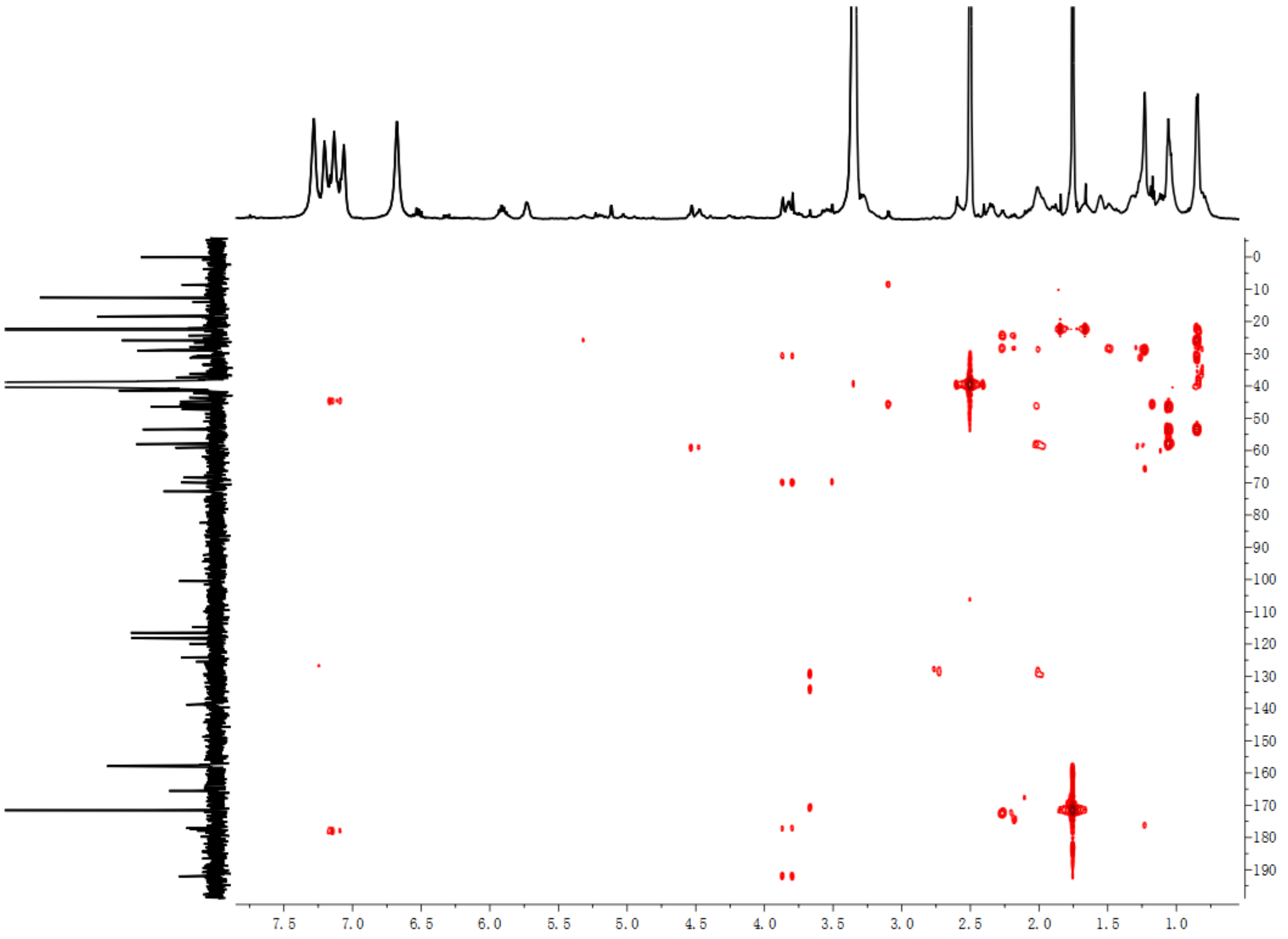

Figure S11. NOESY spectrum of **1** in DMSO-*d6*.

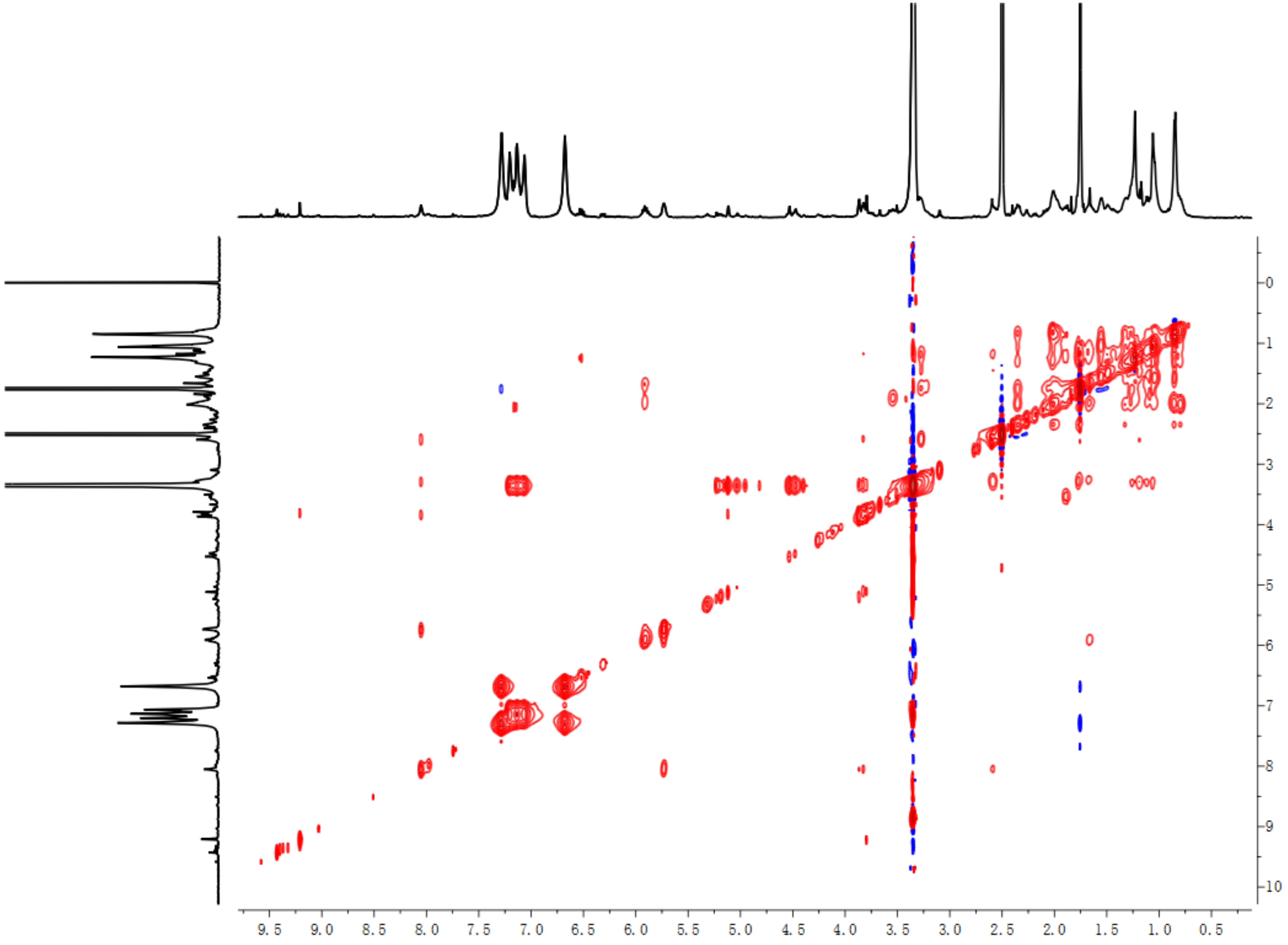

Figure S12. Comparison among the ECD spectra measured for compound **1** and 10-*epi*-HSAF and calculated for (5*S*,6*R*,8*R*,10*R*,11*S*,12*S*,13*R*,14*S*,16*S*,23*S*,25*R*)-**1**.

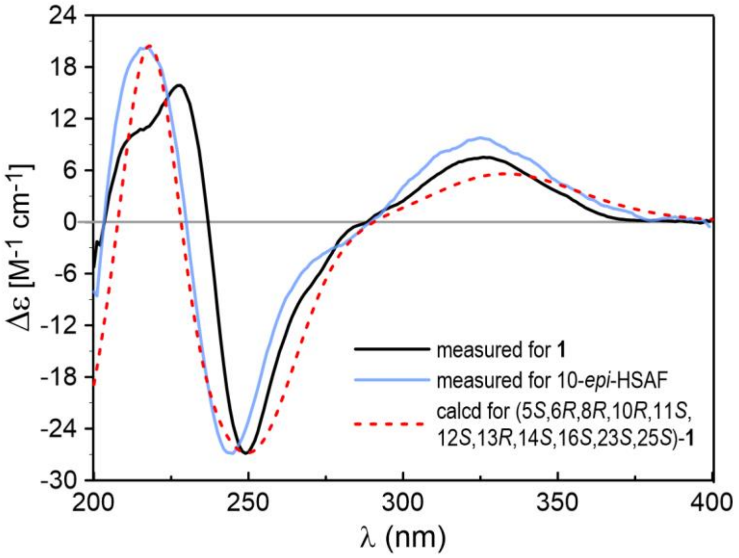

Figure S13. HRESIMS spectrum of **2**.

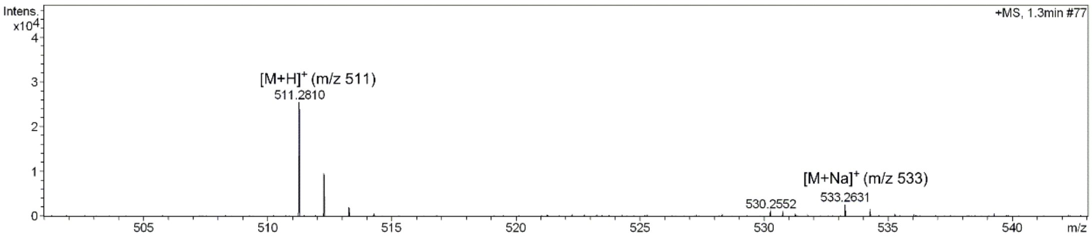

Meas. m/z	#	Ion Formula	Score	m/z	err [ppm]	err [mDa]	mSigma	rdb	e⁻ Conf	N-Rule
511.2810	1	C29H39N2O6	100.00	511.2803	1.5	0.8	26.0	11.5	even	ok
533.2631	1	C29H38N2NaO6	100.00	533.2622	1.7	0.9	54.6	11.5	even	ok
1021.5548	1	C58H77N4O12	100.00	1021.5533	-1.5	-1.5	46.4	22.5	even	ok
1043.5364	1	C58H76N4NaO12	100.00	1043.5352	-1.1	-1.2	135.8	22.5	even	ok

Figure S14. ^{1}H NMR spectrum of **2** in MeOH-*d4*.

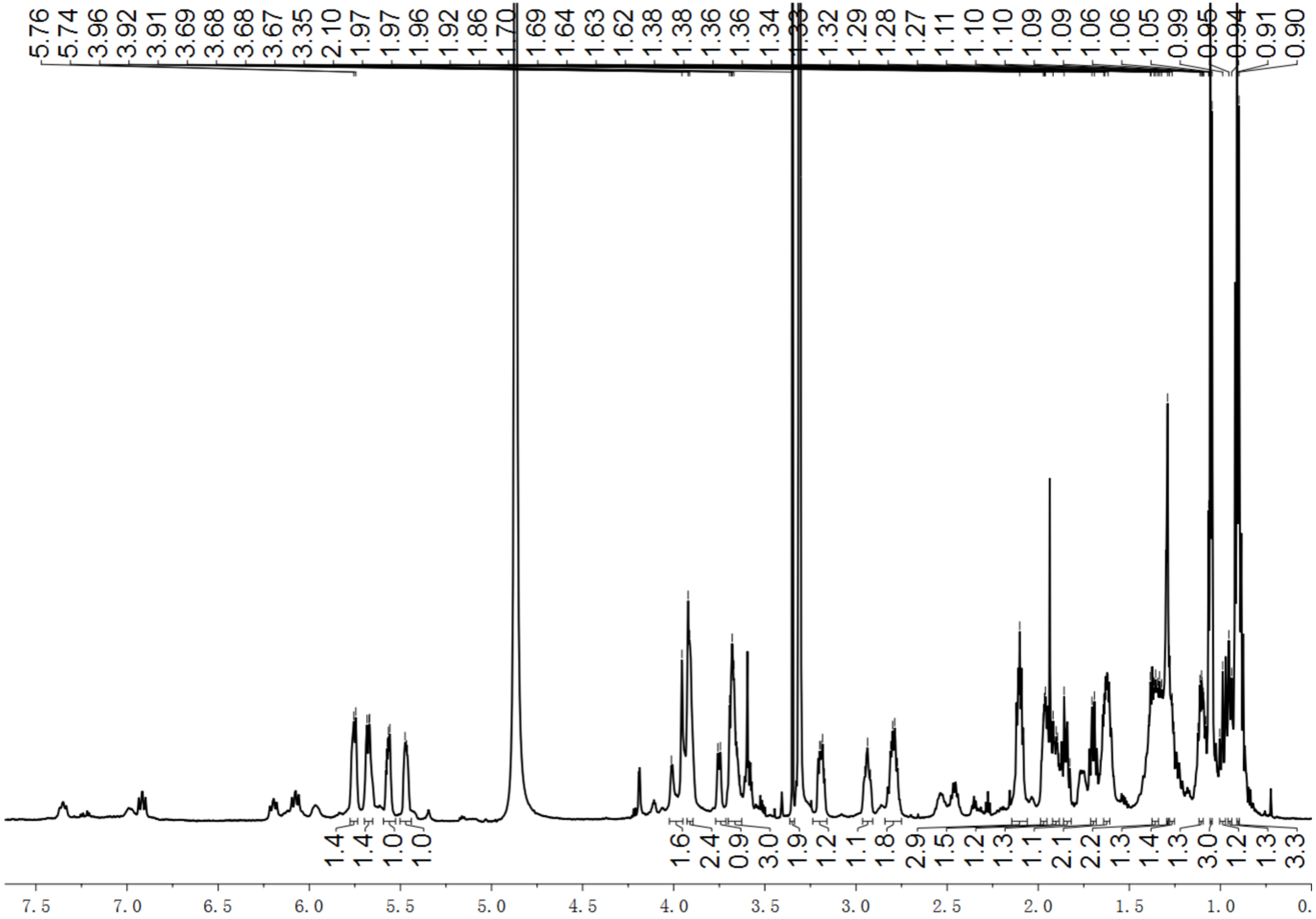

Figure S15. ^{13}C NMR spectrum of **2** in MeOH-*d4*.

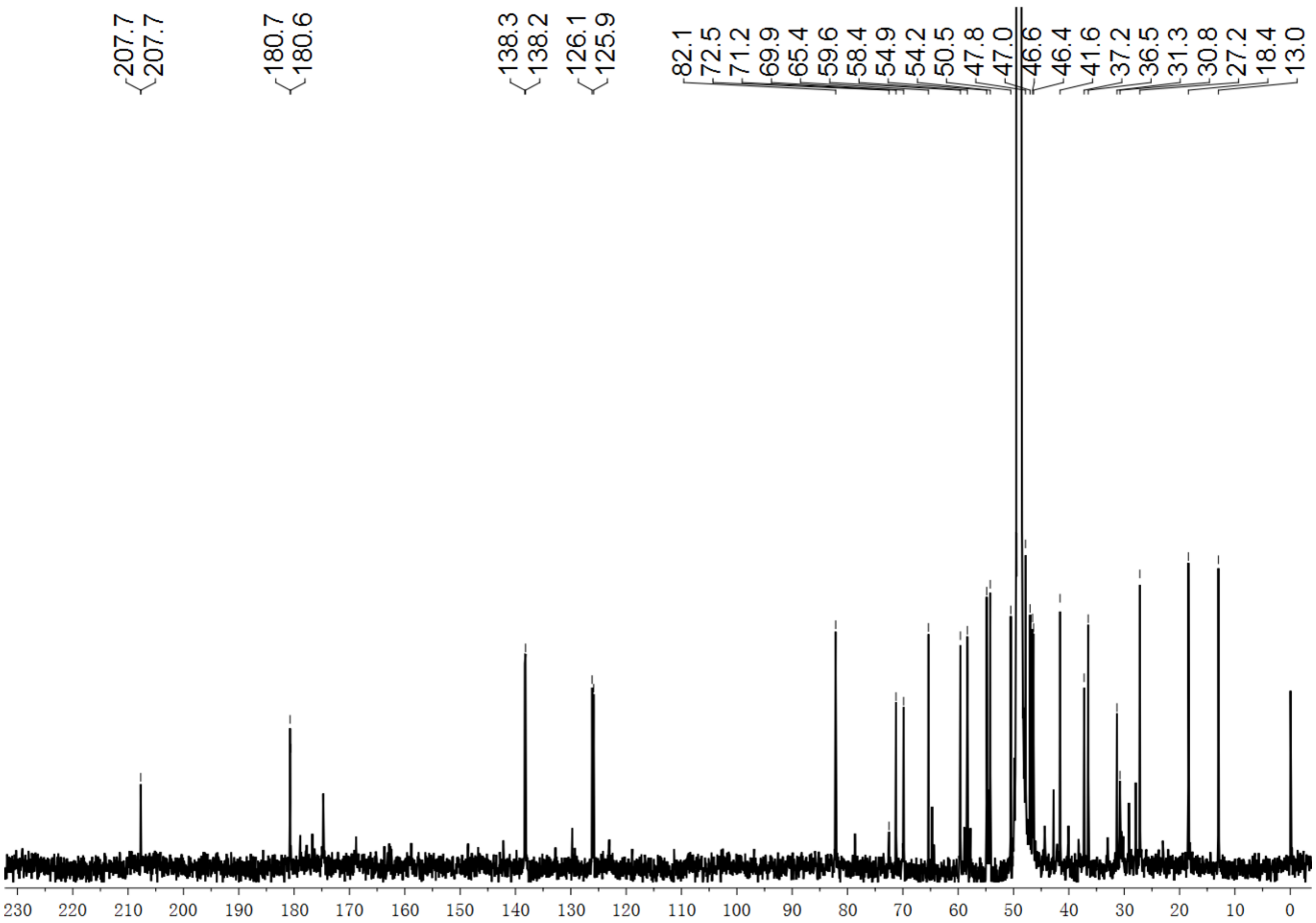

Figure S16. ^{13}C DEPT spectrum of **2** in MeOH-*d4*.

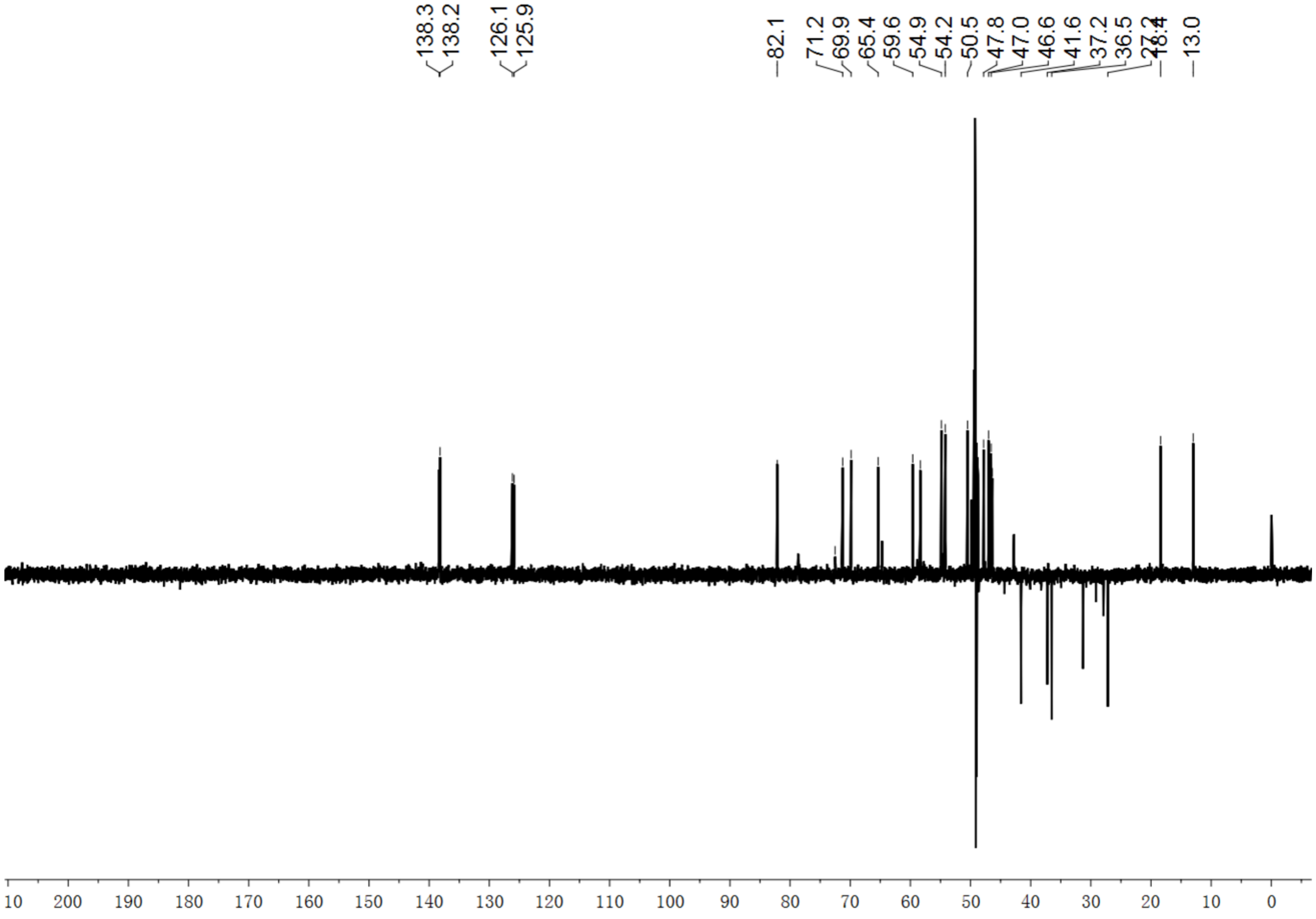

Figure S17. ^{1}H-^{1}H COSY spectrum of **2** in MeOH-*d4*.

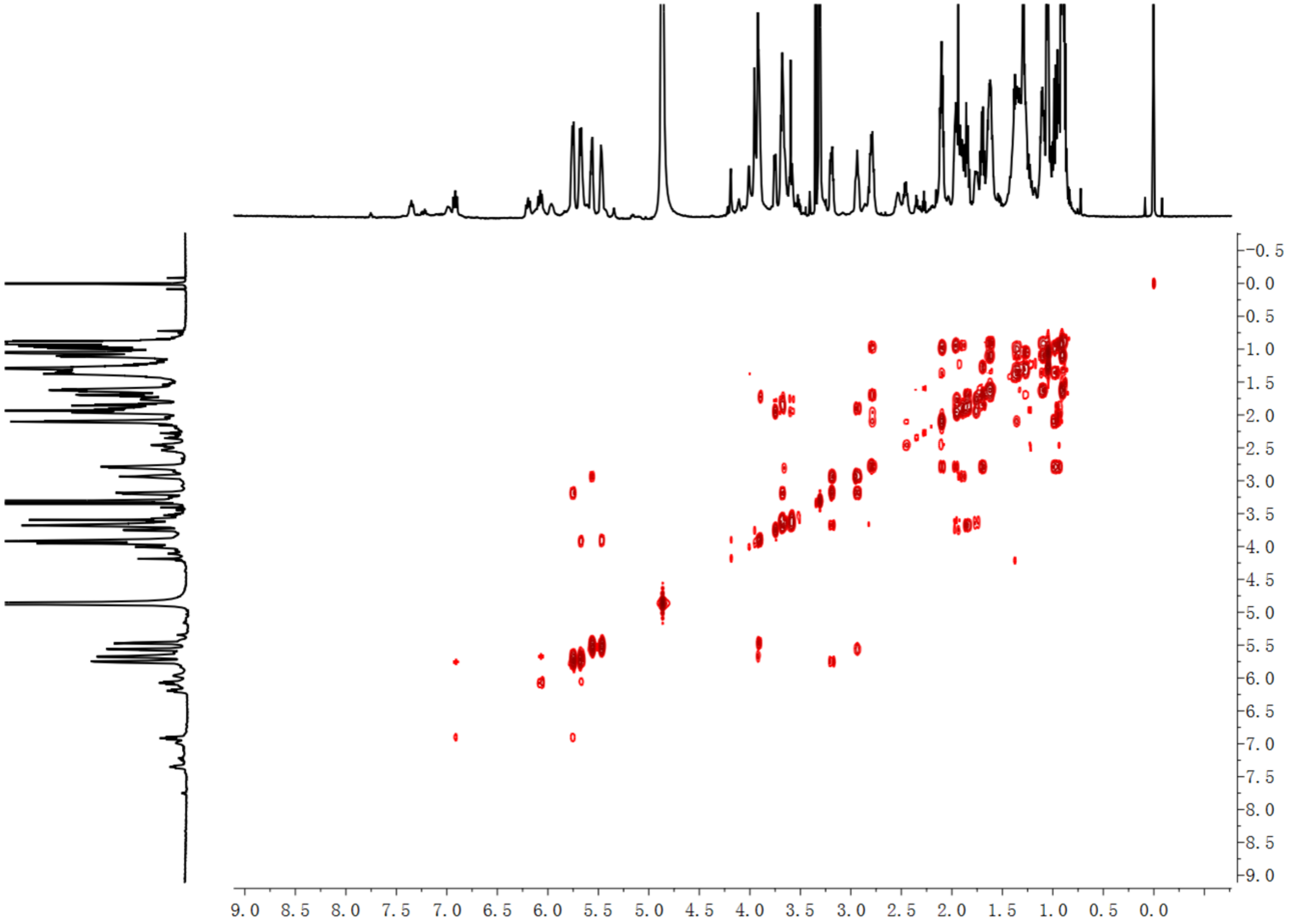

Figure S18. HSQC spectrum of **2** in MeOH-*d4*.

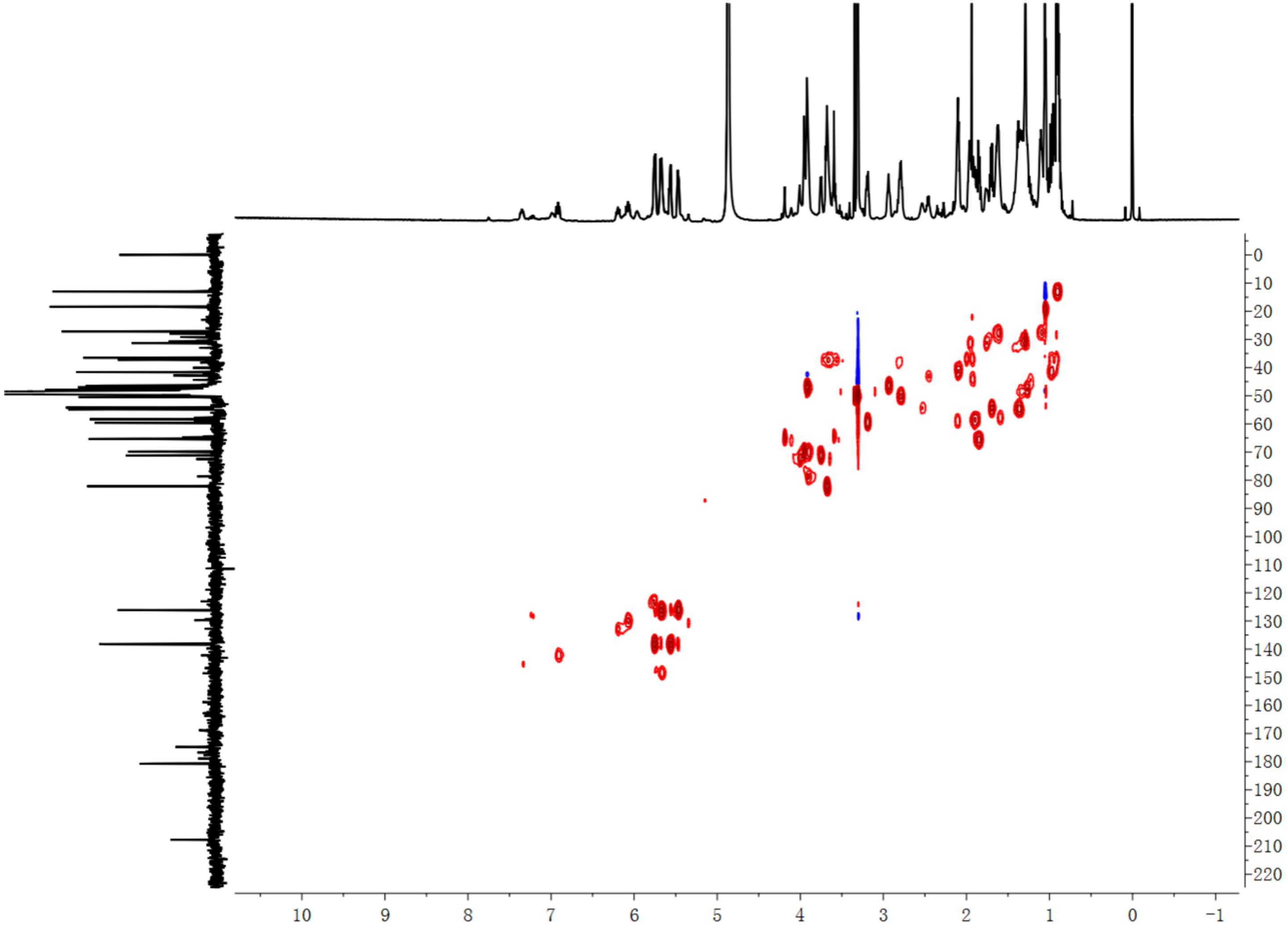

Figure S19. HSBC spectrum of **2** in MeOH-*d4*.

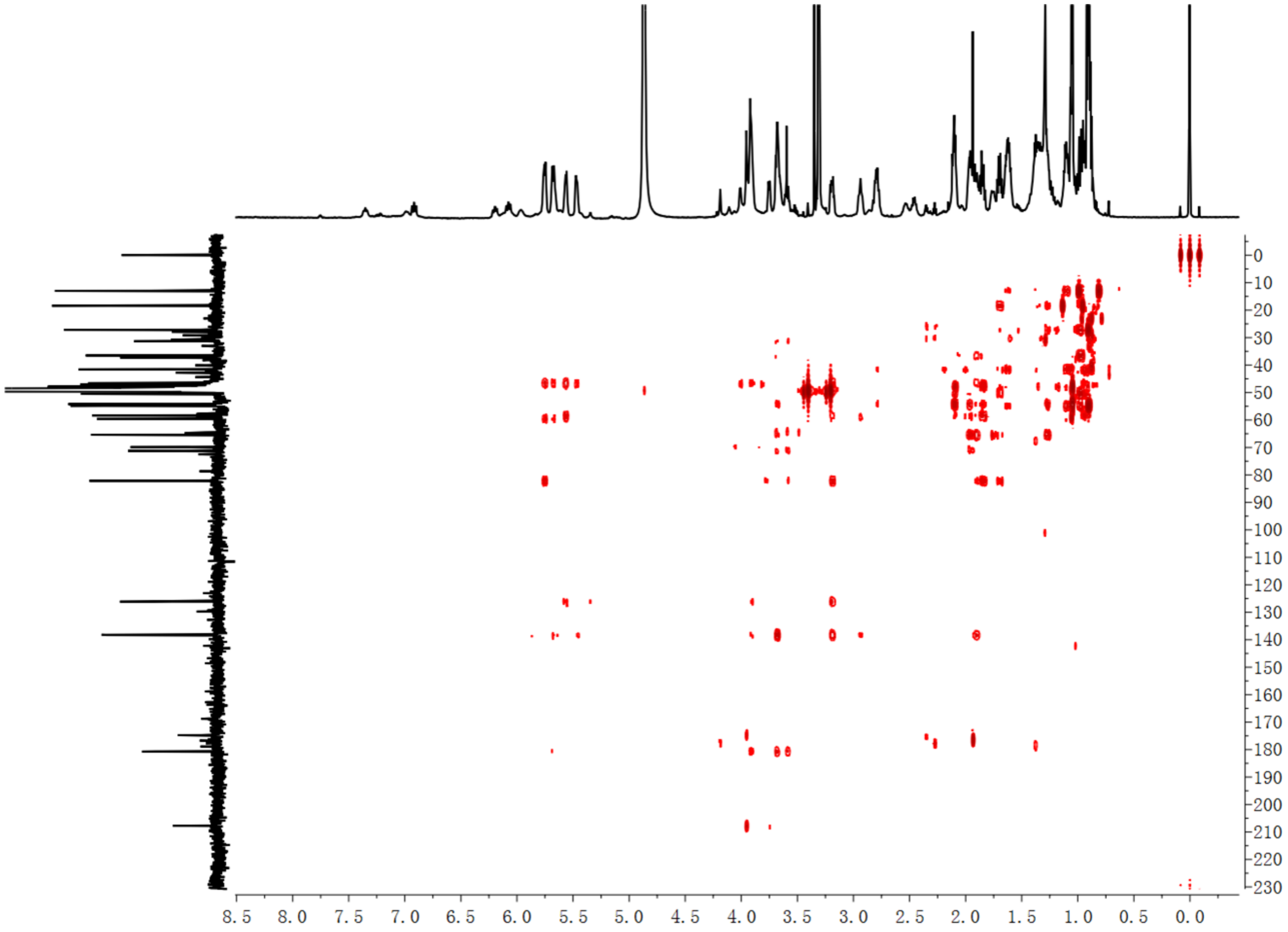

Figure S20. NOESY spectrum of **2** in MeOH-*d4*.

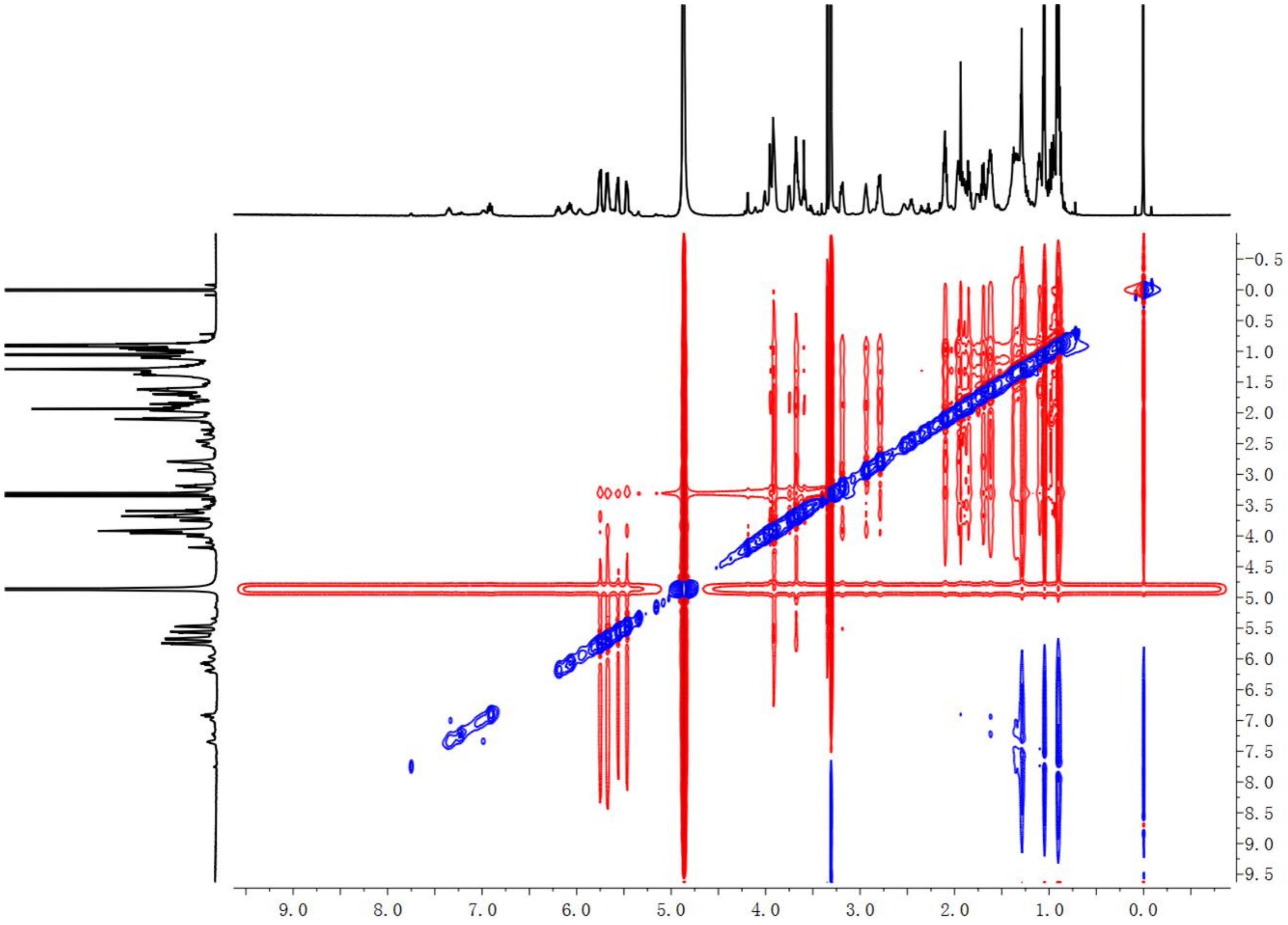

Figure S21. *skoA* disruption in *S. koyangensis* SCSIO 5802AC *via* PCR-targeting.

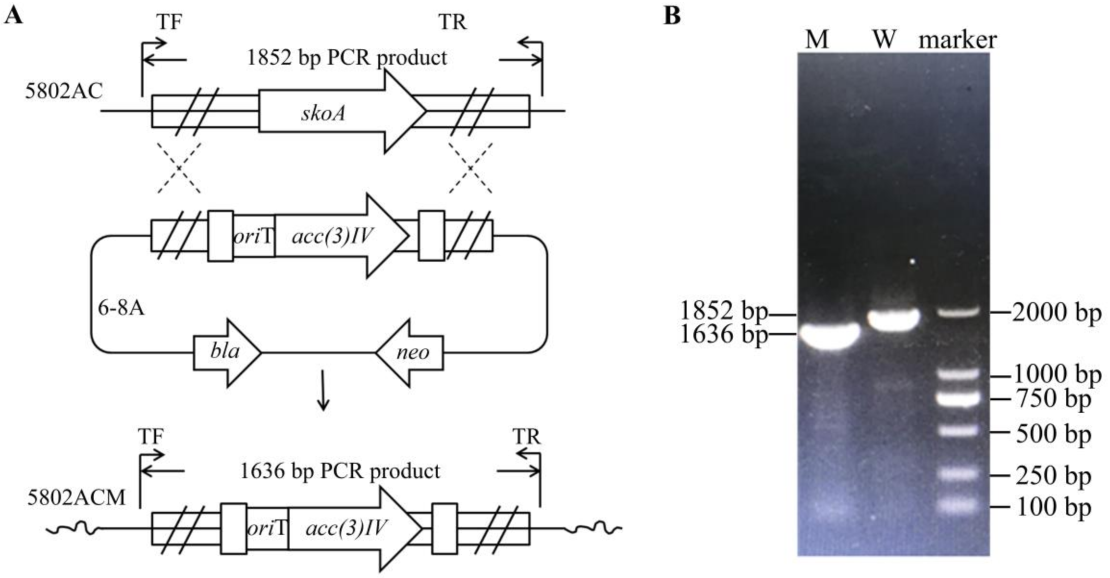

Supplemental References.

[1] MacNeil, D. J.; Gewain, K. M.; Ruby, C. L.; Dezeny, G.; Gibbons, P. H.; MacNeil, T. *Gene* **1992**, *111*, 61-68.

[2] Datsenko, K. A.; Wanner, B. L. *Proc. Natl. Acad. Sci. U.S.A.* **2000**, *97*, 6640-6645.

[3] Gust, B.; Chandra, G.; Jakimowicz, D.; Yuqing, T.; Bruton, C. J.; Chater, K. F. *Adv. Appl. Microbiol.* **2004**, *54*, 107-128.

[4] Paget, M. S. B., Chamberlin, L.; Atrih, A.; Foster,S. J.; Buttner, M. J. J. *Bacteriol.* **1999**, *181*, 204-211.
